# Analysis of Components in Ziziphi Spinosae Semen Before and After Processing Based on Targeted and Untargeted Metabolomics

**DOI:** 10.3390/foods14213771

**Published:** 2025-11-03

**Authors:** Ruiqi Yang, Ze Li, Lulu Dong, Yiran Heng, Lianglei Song, Lijun Guo, Xiangping Pei, Yan Yan, Chenhui Du

**Affiliations:** 1 School of Traditional Chinese Materia Medica, Shanxi University of Chinese Medicine, Taiyuan 030619, China; yangruiqi@sxtcm.edu.cn (R.Y.); leze163@163.com (Z.L.); m13403470315@163.com (L.D.); hengyiran1@163.com (Y.H.); songlianglei2000@163.com (L.S.); guolijun@sxtcm.edu.cn (L.G.); peixp69@163.com (X.P.); 2 Modern Research Center for Traditional Chinese Medicine, Shanxi University, Taiyuan 030006, China

**Keywords:** Fried Ziziphi Spinosae Semen, Ziziphi Spinosae Semen, ^1^H NMR, GC–MS, UHPLC-Q-Exactive Orbitrap-MS

## Abstract

Ziziphi Spinosae Semen (ZSS), a medicinal and edible homologous herbal drug, is commercially available in both raw and fried (FZSS) forms and has been widely used to improve sleep quality. This study aimed to elucidate the differences in chemical composition between the two specifications. A comprehensive metabolomics approach utilizing ^1^H NMR, GC–MS, and UHPLC-Q-Orbitrap-HRMS identified a total of 66 potential biomarkers. The results demonstrated that after frying, the content of fatty acids decreased significantly, while the levels of most primary metabolites (e.g., sugars, amino acids) and secondary metabolites (e.g., alkaloids, flavonoids) increased markedly. Targeted quantification of 14 key components validated these trends: the contents of five fatty acids decreased (*p* < 0.001), whereas the levels of five secondary metabolites (coclaurine, magnoflorine, spinosin, 6‴-feruloylspinosin, and jujuboside A) increased. In contrast, the content of jujuboside B decreased significantly. This study systematically reveals the profound impact of frying on the chemical composition of ZSS, providing a scientific basis for its quality control and processing optimization.

## 1. Introduction

Ziziphi Spinosae Semen (ZSS), derived from the Rhamnaceae family, is the dried and mature seed of the *Ziziphus jujuba* Mill. var. *spinosa* (Bunge) Hu ex H. F. Chou (ZJS). It not only functions as a medication for treating insomnia but also holds edible value. First documented in the renowned ancient Chinese pharmacological work “Shennong Bencaojing” during the Eastern Han Dynasty, ZSS was classified as a high-grade medicinal herb, demonstrating its exceptional therapeutic efficacy. After thousands of years of recorded use, ZSS is currently recognized as one of the most popular traditional Chinese herbal medicines, mainly utilized for calming the mind and improving sleep quality [1]. Studies have shown that ZSS possesses various pharmacological effects, including the treatment of insomnia and anxiety [2], memory modulation, antidepressant effects [3], and anticancer properties [4]. Furthermore, ZSS is a common ingredient in health food products and stands out as one of the few traditional Chinese medicines regulated under the concept of “medicine and food homology” [5].

Herbal medicines are often processed empirically to improve or alter their therapeutic functions. There are two most common commercial specifications of ZSS, including raw Ziziphi Spinosae Semen (ZSS) and Fried Ziziphi Spinosae Semen (FZSS) [6]. Studies have shown that the sedative effect of ZSS is enhanced after frying, making it the most commonly used specification in clinical practice [7,8]. This raises the question: what changes in chemical composition occur before and after frying that lead to alterations in the pharmacological effects of ZSS? Current research primarily focuses on the isolation and identification of chemical components in ZSS [9,10], its in vivo metabolic processes [11], and the mechanisms of action in treating insomnia [12,13] and depression [14,15]. For instance, the main chemical components of ZSS include fatty acids [16], polysaccharides [17], nucleosides [18], and amino acids [19]. Among them, there are as many as 41 types of fatty acids [20], accounting for over 30% of the raw material [19]. Polysaccharides are another major component, constituting approximately 1.05% of ZSS and identified primarily as acidic heteropolysaccharides [21]. Additionally, ZSS seeds contain more than 22 amino acids (0.42%) and 11 nucleosides (0.02%) [19,22]. However, there are still few reports on how the chemical constituents of ZSS change before and after processing.

Untargeted metabolomics combined with chemometrics has been demonstrated to be a powerful tool for comprehensive analysis of metabolites in drugs and foods [23,24]. The most commonly used tools in metabolomics research include nuclear magnetic resonance (NMR), gas chromatography mass spectrometry (GC–MS), and liquid chromatography mass spectrometry (LC–MS) [23,25]. Among them, GC–MS is a robust and reproducible technique, frequently employed for the detection of volatile components or for the analysis of fatty acid-based compounds (after derivatization) [26]. In contrast, LC–MS offers a broad analytical range and high sensitivity, enabling the analysis of compounds with various polarities and molecular weights, despite relatively poor reproducibility. NMR spectroscopy provides excellent reproducibility, albeit with lower sensitivity [27]. Considering the strengths and limitations of these techniques, it is essential to develop a comprehensive metabolomics strategy combining both untargeted and targeted approaches to thoroughly analyze the differences in primary and secondary metabolites between ZSS and FZSS.

In this study, we employed untargeted metabolomics approach utilizing ^1^H NMR, GC–MS, and LC-MS platforms to comprehensively analyze the chemical constituents of (ZSS) during its processing. The change patterns of these components were analyzed, and differential metabolites were identified by applying variable importance in projection (VIP) scores and S-plots. Subsequently, targeted metabolomics was employed for the quantitative analysis of key differential compounds using authentic standards, to elucidate their transformation patterns and establish quantitative relationships during the processing of ZSS. This work could help understand the biochemical changes in ZSS induced by processing and could further provide a comprehensive scientific basis for its quality control.

## 2. Materials and Methods

### 2.1. Chemicals and Reagents

Standards of coclaurine, magnoflorine, vicenin II, spinosin, kaempferol-3-O-rutinoside, 6‴-feruloylspinosin, jujuboside A, betulinic acid, quercetin, ceanothic acid, vitexin and apigenin, were purchased from Baoji Herbest Bio-Tech Co. (Baoji, Shaanxi, China). Swertisin was obtained from Sichuan Weikeqi Biotechnology Co. (Chengdu, Sichuan, China). Isovitexin was obtained from Chengdu Ruifensi Biotechnology Co. (Chengdu, Sichuan, China). Jujuboside B was purchased from Nanjing Spring & Autumn Biological Engineering Co. (Nanjing, Jiangsu, China).

Palmitic acid, 9,12-Octadecadienoic acid, 9-Octadecenoic acid, stearic acid and eicosanoic acid were purchased from Tokyo Chemical Industries Co. Ltd. (Tokyo, Japan). Heptadecanoic acid (internal standard), squalene and γ-tocopherol were purchased from the Aladdin Chemical Co. (Shanghai, China). All standards were ≥98% pure.

The following reagents were acquired from their respective suppliers: acetonitrile (MS grade) from Thermo Fisher Scientific (Waltham, MA, USA); formic acid (MS grade), methanol-d4, and bistzfrifluoroacetamide (BSTFA, with 1% TMCS) from Sigma-Aldrich (St. Louis, MO, USA). Double-deionized water was produced with a Milli-Q system (Millipore Corporation, Billerica, MA, USA), and all other chemicals were of analytical grade. ZSS (210191015) was provided by Shanxi Zhendong Chinese Herbal Development Co. (Changzhi, China), and authenticated by Prof. Chenhui Du as the dried seeds of *Ziziphus jujuba* Mill. var. *spinosa* (Bunge) Hu ex H.F. Chou according to the Chinese Pharmacopoeia (2020 version). The voucher specimens were preserved at the department of Chinese Medicine Resources and Identification, Shanxi University of Chinese Medicine, Jinzhong, China. FZSS samples were prepared by stir-firing in oil bath of 170 °C for 5 min to become puffy and slightly darker in color [28].

### 2.2. Color Measurements

Color measurements were carried out for the ZSS and FZSS using a Chroma Meter (NH310, Shenzhen Threenh Technology Co., Shenzhen, China). The L*, a*, b* color space defines color attributes using three parameters: L* represents lightness (100) to darkness (0), a* indicates the red-green axis (positive for red, negative for green), and b* denotes the yellow-blue axis (positive for yellow, negative for blue). The L*, a*, and b* values of ZSS and FZSS were measured, with three replicates for each treatment.

### 2.3. Untargeted Metabolomics Analysis

#### 2.3.1. ^1^H NMR Analysis

Sample preparation: Sample preparation was performed as previously described [29]. Briefly, the tested samples were ground into powder. About 0.2 g of the finely ground powder was accurately weighed into a 10 mL glass centrifuge tube. Then, 6 mL of CHCl_3_-MeOH-H_2_O (2:1:1, *v*/*v*) was added, the mixture was vortexed for 1 min and sonicated for 25 min. After centrifugation at 3500 rpm for 25 min, the supernatant (the polar phase) was collected and dried. The residue was finally dissolved in 400 μL of CD_3_OD (adjusted to pH 6.0 with NaOH) containing 0.1% TSP. Subsequently, 600 μL of this solution was transferred into a 5 mm NMR tube for analysis.

Test conditions: The ^1^H NMR spectra were recorded on a Bruker 600 MHz AVANCE III NMR spectrometer (Bruker, Bremen, Germany). CD_3_OD was used for internal locking purposes. Each ^1^H NMR spectrum was acquired using the Noesygppr1D pulse sequence, which consisted of 64 scans requiring 5 min of acquisition time with the following parameters: digital resolution = 0.188 Hz/point, pulse width = 30°, and relaxation delay = 1.0 s. The ^1^H NMR spectra were processed using MestReNova 14.0 software (Mestrelab Research, Santiago de Compostela, Spain). After phase and baseline correction, the spectra were internally referenced to the TSP signal set at *δ* 0.00. All spectral regions between *δ* 0.6 and 9.5 were divided into regions with widths of 0.01 ppm. The regions of *δ* 4.77–5.03 and *δ* 3.30–3.37 were excluded from the analysis due to the presence of residual signals corresponding to D_2_O and CD_3_OD, respectively. Furthermore, two-dimensional NMR (^1^H–^1^H COSY) spectroscopy was employed to support the assignment and confirmation of the secondary metabolites.

#### 2.3.2. GC–MS Analysis

Sample preparation: ZSS and FZSS fatty oil samples were prepared according to our previous study with some modifications [30]. Briefly, the tested samples were ground into powder and passed through a 60-mesh (0.3 mm) sieve. Approximately 2.0 g of the sample powder was placed in a soxhlet apparatus and extracted with 90 mL of petroleum ether (40–60 °C) for 4 h. Then, anhydrous Na_2_SO_4_ powder was added to remove residual moisture, and the solution was filtered. The solvent was evaporated under reduced pressure to obtain the fatty oil. Subsequently, 50 μL of the fatty oil was silylated by reacting with 150 μL of BSTFA (containing 1% TMCS). The mixture was incubated at 90 °C under continuous shaking (300 rpm) for 90 min. After incubation, the mixture was cooled to 25 °C and passed through a 0.22 μm membrane filter prior to injection into the GC system. Quality Control (QC) samples were prepared by mixing equal volumes of ZSS and FZSS samples to enable the reproducibility of the mass spectrometric results to be assessed.

GC–MS analysis of fatty acids was performed on an Agilent 7890B gas chromatography instrument equipped with Agilent 5977B Mass Selective Detector (MSD), using an HP-5MS capillary column (30 m × 0.25 mm × 0.25 μm, Agilent Technologies, Santa Clara, CA, USA). The analytical procedure was performed according to our previous study [29]. Helium was employed as the carrier gas at a constant flow rate of 1 mL/min. The GC temperature program was optimized as follows: initial column temperature was held at 150 °C for 1 min, then ramped at 5 °C/min to 215 °C (held for 5 min) followed by an increase at 5 °C/min to 240 °C (held for 1 min), and finally raised at 10 °C/min to 270 °C (held for 10 min). A solvent delay of 4 min was set. Mass spectrometry was performed in full-scan mode with electron impact ionization (70 eV), covering a mass range of 30–500 *m*/*z*. The ion source, injector, and transfer line temperatures were maintained at 200 °C, 250 °C, and 280 °C, respectively.

#### 2.3.3. UPLC-MS Analysis

Sample preparation: After being extracted with the soxhlet apparatus, the residues from ZSS and FZSS were transferred into a round-bottom flask. Then, 20 mL of 70% methanol was added, and the mixture was extracted under reflux for 2 h. The resulting solution was filtered and evaporated to dryness. The dried extract was reconstituted in methanol and diluted to a final volume of 10 mL. The solution was then filtered through a 0.22 μm membrane filter prior to UPLC-MS analysis. QC samples were prepared by mixing equal volumes of ZSS and FZSS samples to enable the reproducibility of the mass spectrometric results to be assessed.

For UHPLC-Q-Exactive Orbitrap-MS/MS analysis, the extracts of ZSS and FZSS were analyzed using a Thermo Scientific™ Q Exactive Plus mass spectrometer (Thermo Scientific, Bremen, Germany). Chromatographic separation was performed on U3000 system equipped with a Waters Acquity UPLC HSS T3 column (150 mm × 2.1 mm, 1.8 μm). The separation used a mobile phase of 0.1% formic acid in water (A) and acetonitrile (B) with a flow rate of 0.3 mL/min. The gradient elution program was: 0–8 min, 5–17% B; 8–10 min, 17%; 10–11 min, 17–18%; 11–12 min, 18–20%; 12–22 min, 20–33%; 22–25 min, 33–100%; 25–27 min, 100%. The column and auto-sampler temperatures were maintained at 40 °C and 4 °C, respectively.

The Full MS-dd MS^2^ Scan Was Separately Performed in Both Positive Ion and Negative Ion Modes. The MS parameters for the positive ion mode were as follows: spray voltage, 3.5 kV; sheath gas flow rate, 40 arb; auxiliary gas flow rate, 10 arb; capillary temperature, 320 °C; and vaporizer temperature, 350 °C. The full MS scan range was *m*/*z* 150–1500 with a resolution of 70,000. The dd-MS^2^ resolution was 17,500. For the negative ion mode, the spray voltage was set to 3.2 kV, the mass range was *m*/*z* 100–1500, and other parameters were identical to those used in the positive ion mode. The raw LC-MS data were processed using Xcalibur 4.1 software (Thermo Fisher Scientific, Waltham, MA, USA). Peak extraction, deconvolution, alignment, and normalization were performed with Compound Discoverer (CD) 3.3 software, resulting in a three-dimensional data matrix containing sample ID, retention time (tR)-*m*/*z*, and peak intensities. The key processing parameters were set as follows: maximum mass error of 5 mDa; double-bond equivalent (DBE), 0–50; election count, odd; maximum H deficit, 6; fragment number of bonds, 4. For multivariate statistical analysis, the resulting data matrix was processed using SIMCA-P (version 14.1, Umetrics, Umea, Sweden). Metabolite identification was performed by comparing the acquired MS/MS spectra with those of reference standards in the mzCloud and ChemSpider databases.

### 2.4. Targeted Metabolomics Based on GC–MS

#### 2.4.1. Sample Preparation

The fatty oil was dissolved in n-hexane and made up to a final volume of 5 mL. Then, a 50 μL mixture was transferred to a tube, followed by the addition of 10 μL of the internal standard solution and 180 μL of silylation reagent. After vortexing for 1 min, the mixture was silylated according to the procedure described in Section 2.3.2.

#### 2.4.2. GC–MS Conditions

For quantitative analysis, the mass spectrometer was operated in selected ion monitoring (SIM) mode with an electron impact ionization energy of 70 eV and a mass scan range of 50–500 *m*/*z*, with all other conditions as per Section 2.3.2. The developed method was validated to be precise, accurate, and sensitive for the simultaneous determination of all seven target fatty acids in ZSS and FZSS samples. A standard curve was established using a series of concentrations of a standard mixture containing palmitic acid, stearic acid, 9-octadecenoic acid, 9,12-octadecadienoic acid, eicosanoic acid, squalene, and tocopherol for the quantification of these seven compounds.

#### 2.4.3. Method Validation

The qualitative and quantitative ions for selected ion monitoring (SIM) mode were selected based on the most abundant and characteristic ions observed in full scan mode. Each compound was identified by comparing its mass spectrum and relative retention index with those in the NIST 14 database. The identity was further confirmed by matching the retention time with that of an authentic reference standard. The content of each compound was calculated using MassHunter Quantitative Analysis 10.2 software(Agilent Technologies, Santa Clara, CA, USA).

### 2.5. Quantification of Secondary Differential Metabolites by HPLC-DAD-ELSD

Quantitative analyses were performed on a Waters 2695 HPLC system equipped with a 2998 DAD detector and a 6000 ELSD detector. Seven different secondary metabolites were targeted for quantification. The samples used were the same as those analyzed by UPLC-MS. Chromatographic separation of the seven components was achieved using an Apollo C18 column (250 mm × 4.6 mm, 5 μm) maintained at 30 °C, with a flow rate of 1.0 mL/min. The mobile phase consisted of 0.1% formic acid in water (A) and acetonitrile (B), using the following gradient program: 0–26 min, 10–20% B; 26–30 min, 20–23% B; 30–43 min, 23–26% B; 43–45 min, 26–37% B; 45–47 min, 37% B; 47–54 min, 37–39% B; 54–63 min, 39–100% B. The injection volume was 10 μL. Detection was carried out at wavelengths of 227 nm and 335 nm. The ELSD parameters were set as follows: drift tube temperature at 105 °C and nitrogen gas flow rate at 2.5 L/min.

A standard curve was constructed using a series of concentrations of a standard mixture containing coclaurine, magnoflorine, vicenin II, spinosin, 6‴-feruloylspinosin, jujuboside A, and jujuboside B. Calibration curves for the nitrogen-containing compounds and flavonoids were generated by plotting the peak area against the concentration of each analyte. For jujuboside A and jujuboside B, the calibration curves were established by plotting the logarithm of the peak area against the logarithm of the concentration. All compounds exhibited excellent linearity within their respective concentration ranges, with correlation coefficients (r) greater than 0.9991.

### 2.6. Statistical Analysis

Multivariate data analysis, including principal component analysis (PCA) and orthogonal partial least squares-discriminant analysis (OPLS-DA), was performed using SIMCA-P software(version 14.1, Umetrics, Umea, Sweden). The OPLS-DA model was validated by a permutation test (200 permutations) and its significance was assessed by sevenfold cross-validation analysis of variance (CV-ANOVA). Variables with a VIP value greater than 1.0 in the OPLS-DA model and a *p* value less than 0.05 in Student’s *t*-test were selected as potential chemical markers. Subsequently, these filtered compounds were subjected to heatmap visualization and hierarchical clustering analysis (HCA) using MetaboAnalyst 3.0 to identify relatively homogeneous sample clusters and illustrate metabolite content changes across different sample groups.

All data are presented as the mean ± standard deviation (SD). Differences between groups were analyzed using Student’s *t*-test. A *p* value of less than 0.05 was considered statistically significant. Box plots illustrating the relative contents of potential biomarkers were generated using GraphPad Prism software (Version 7.0, San Diego, CA, USA). All statistical analyses were performed with IBM SPSS Statistics 26.0, while the figures were designed using GraphPad Prism 6.0.

## 3. Results and Discussion

### 3.1. Color Measurement Analysis

**Figure 1:**
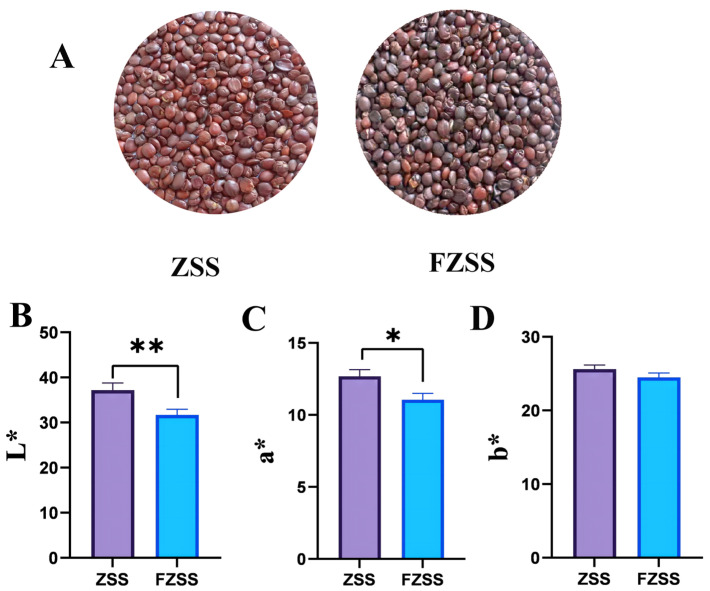
Color analysis of ZSS and FZSS seeds. (**A**) Representative photographs of ZSS and FZSS seeds. (**B**–**D**) Quantitative analysis of seed color parameters (L*, a*, b*) measured by a portable colorimeter. Statistically significant differences between ZSS and FZSS were determined by *t*-test (* *p* < 0.05, ** *p* < 0.01).

The variations in the coat color of ZSS reflect changes in its chemical composition. As shown in Figure 1A, raw ZSS exhibits a reddish-brown color, which darkens to a deep brown after processing. This darkening was usually attributed to the formation of brown pigments via Maillard-type non-enzymatic reactions between reducing sugars and free amino acids or amines [31].

As shown in Figure 1B–D, the color parameters (L*, a*, b*) of the samples changed after processing. The lightness (L*), which represents the white-black axis, decreased significantly on the FZSS coat, indicating a darkening effect. The a* values (red-green axis) of FZSS showed a significant increase (*p* < 0.05), suggesting a reduction in redness. Notably, no significant difference was observed in the b* values (yellow-blue axis) between FZSS and ZSS, indicating there was no significant change in yellowness.

### 3.2. Results of Untargeted Metabolomics Analysis

In this study, we employed three analytical platforms: ^1^H NMR for the identification and relative quantification of low molecular weight primary metabolites, GC–MS for key fatty acid metabolites, and UPLC-MS for secondary metabolites. By integrating these platforms, we systematically and comprehensively characterized the metabolic profiles of ZSS and FZSS.

#### 3.2.1. ^1^H NMR Profiles and Characterization of Metabolites of ZSS and FZSS

**Figure 2:**
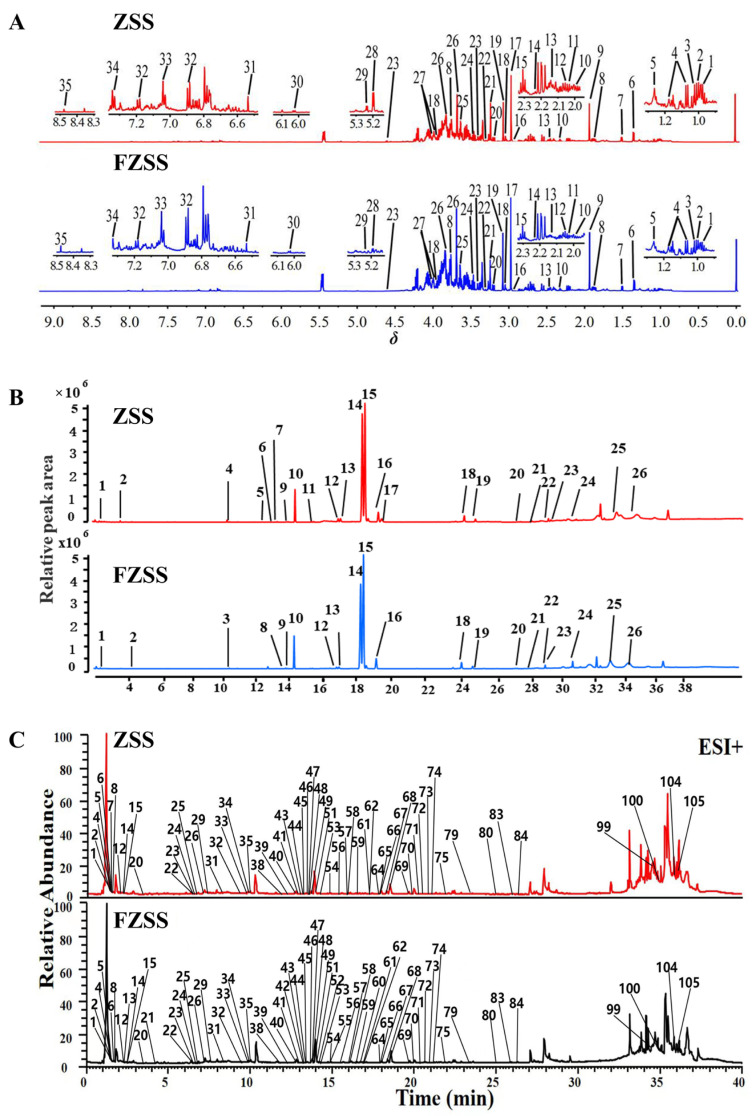
(**A**) Representative ^1^H NMR spectra of ZSS and FZSS. Peak assignments are provided in Table S1. (**B**) GC–MS total ion chromatograms (TICs) of the TMCS derivatives of fatty oils from ZSS and FZSS. Compound identities are provided in Table S2. (**C**) UPLC-MS total ion chromatograms (positive ion mode) of ZSS and FZSS. Compound identities are provided in Table S3.

^1^H NMR was used to characterize the primary metabolites in ZSS and FZSS. Representative ^1^H NMR spectra of the ZSS and FZSS extractions are shown in Figure 2A. They showed little difference in chromatograms, while peak intensities varied. A total of 35 metabolites were unambiguously assigned by comparing their chemical shifts in standard references, ^1^H-^1^H COSY spectra, and databases (Human Metabolome Database (http://www.hmdb.ca/, accessed on 15 March 2025) and PubChem Database (http://pubchem.ncbi.nlm.nih.gov/, accessed on 15 March 2025). The detailed information is listed in Table S1. The ^1^H NMR spectra were divided into three different regions: the *δ* 0–3.0 ppm region, which exhibited high-intensity signals from amino acids and organic acids; the *δ* 3.0–5.5 ppm region, dominated by sugars and sugar alcohols; and the *δ* 5.5–9.5 ppm region, characteristic of aromatic compounds.

Notably, visual inspection of the spectra revealed that the signal intensities of the following metabolites were stronger in FZSS than in ZSS: formic acid [*δ* 8.44 (s)], *γ*-aminobutyric acid (GABA) [*δ* 2.31 (t, 7.20)], creatinine [*δ* 3.04 (s)], saccharose [*δ* 3.66 (s), 3.81 (m)], tyrosine [*δ* 6.90 (d, 7.80), 7.19 (d, 8.40)], and *α*-glucose [δ 5.23 (d, 3.60)]. In contrast, the signal intensity of xylose [*δ* 5.20 (d, 3.60)] was lower in FZSS. Furthermore, the spectra revealed a variety of other metabolites, including organic acids, cholines, and nucleosides.

To resolve signal overlap observed in the 1D-NMR spectra, a set of 2D NMR spectroscopic experiments Viz. ^1^H-^1^H COSY was employed to assist in the assignment of metabolites in ZSS and FZSS, and in comparison, to reference standards and chemical shifts reported in the literature (Figure S1). By analyzing the 2D NMR spectra processed with MestReNova and comparing the signals with those of standards, several secondary metabolites were identified. Specifically, the chemical attribution of magnoflorine was determined at *δ* 2.94, the chemical attribution of 6‴-feruloylspinosin was determined at *δ* 3.74, *δ* 7.07 and *δ* 7.60, and the chemical attribution of spinosin was determined at *δ* 3.10 and *δ* 7.96. The analysis identified jujuboside A, betulinic acid, magnoflorine, 6‴-feruloylspinosin and spinosin, full of them confirmed by isolation and full spectroscopic/spectrometric assignment using nuclear magnetic resonance (NMR).

#### 3.2.2. GC–MS Profile and Characterization of Fatty Acids

A previous study showed that fatty oil constitutes over 30% of the total weight of ZSS [32]. The most important components of fatty oils are polyunsaturated fatty acids (PUFAs), particularly omega-6 (n-6) PUFAs. In the central nervous system, PUFAs play critical roles in neurogenesis, brain development, synaptic formation, and neuroinflammation.

Linoleic acid (LA, C18:2n-6), a major fatty acid in ZSS, is an essential n-6 PUFA and a precursor to arachidonic acid (AA, C20:4n-6). A recent study have implicated LA in neurodevelopmental, neurophysiological and metabolic syndrome (MetS) processes. Moreover, its oxidized metabolites are known modulators of pain and inflammatory responses [33].

ZSS and FZSS were analyzed by GC–MS after derivatization, and their typical total ion chromatograms (TIC) are presented in Figure 2B. The number and abundance of metabolite peaks showed slight differences between ZSS and FZSS, which was consistent with the ^1^H NMR findings. Based on comparison with the NIST mass spectral database, a total of 39 metabolites were putatively identified and classified into two categories: fatty acids and their derivatives, and terpenes. Among these, 28 components were common to both ZSS and FZSS. The detailed identification information is listed in Table S2.

- Fatty Acids and Derivatives;

A statistical analysis was conducted on fatty acids and their derivatives. A total of 13 fatty acids and 19 fatty acid derivatives were detected in this study. The unsaturated fatty acids (UFAs) in ZSS accounted for 79.44% of the total fatty acids, while in FZSS, UFAs accounted for 48.02%. After processing, the overall levels of fatty acids and their derivatives in ZSS changed significantly. The proportion of fatty acid derivatives in processed ZSS increased from 3.62% to 6.17% (Table S2).

Interestingly, it was found that three fatty acids and four fatty acid esters are unique to ZSS and are not present in FZSS. These compounds are: nonanoic acid, myristic acid, pentadecanoic acid, 1,2-benzenedicarboxylic acid, butyl 2-methylpropyl ester, 2-chloroethyl linoleate, 9,15-octadecadienoic acid, methyl ester, (Z,Z)-, and trans-13-octadecenoic acid, methyl ester. The absence of these fatty acid esters in FZSS is likely due to the cleavage of ester bonds during the high-temperature processing.

- Terpenes and other compounds;

A total of 2 terpene components were detected and identified, including squalene and Lup-20(29)-en-3-ol, 28-al, (3β)-. The relative content of terpene components increased significantly from 3.93% to 9.58% after processing.

In addition to the structures mentioned above, trace amounts of other components, such as sucrose and the vitamin compound gamma-tocopherol, were detected in roasted date seeds. The content of gamma-tocopherol, a major antioxidant in plant cells, increased from 0.97% to 3.18% after processing.

#### 3.2.3. Identification and Characterization of Metabolites Based on LC-MS

An untargeted UPLC-Q-Exactive-MS/MS based metabolomics analysis was performed to profile the differential metabolites between ZSS and FZSS. The base peak chromatograms (BPC) of both samples are shown in Figure 2C and Figure S2. A total of 108 metabolites were detected across both positive and negative ionization modes. The identities of these compounds were further confirmed by analyzing fragment ions and comparing them with reference standards or literature data. Detailed mass spectrometry information for the identified components—including retention time, calculated and observed *m*/*z*, mass error, molecular formula, and MS/MS spectra—is provided in Table S3.

### 3.3. Multivariate Statistical Analysis

#### 3.3.1. ^1^H NMR Data Analysis

**Figure 3:**
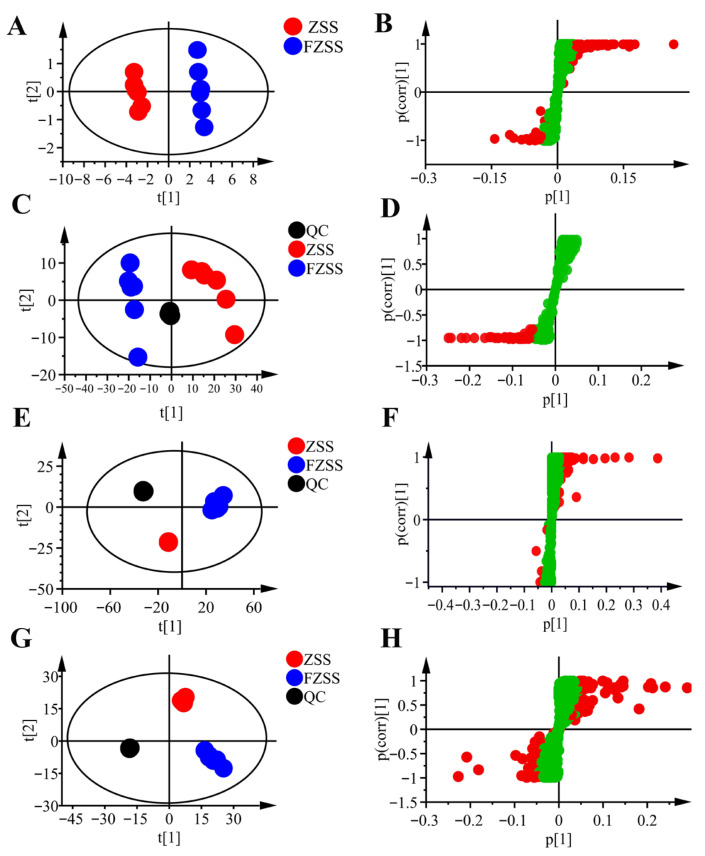
PCA score plots and S-plots comparing ZSS and FZSS (N = 6). (**A**,**B**) Primary metabolites (^1^H NMR). (**C**,**D**) Fatty oils (GC–MS). (**E**,**F**) Secondary metabolites, LC-MS positive mode. (**G**,**H**) Secondary metabolites, LC-MS negative mode. (In S-plots, red points indicate VIP > 1).

In the unsupervised PCA score plot (Figure 3A), a clear separation between the ZSS and FZSS groups was observed along principal component 1 (PC1, 80.20%) and principal component 2 (PC2, 7.91%), indicating that the processing significantly altered the metabolic profile of FZSS compared to ZSS. To identify potential characteristic components distinguishing the two groups, a supervised OPLS-DA model was established. The validity of the model was confirmed by a permutation test (200 permutations; Figure S3A,B), where the resulting *R*^2^ and *Q*^2^ values of all permuted models were lower than the original points on the right. Additionally, the regression line of *Q*^2^ had a negative intercept, suggesting that the model was not overfitted. In the corresponding S-plot, each point represents a metabolite.

Finally, 16 metabolites were selected as potential biomarkers for distinguishing between ZSS and FZSS based on the criteria of VIP > 1.0 and *p* < 0.05. These biomarkers comprised 8 amino acids and nitrogen-containing compounds (e.g., lysine, proline, glutamine, creatine, choline), 3 sugars (e.g., fructose, sucrose), 2 organic acids and their derivatives (e.g., acetic acid, acetoacetic acid), and 3 secondary metabolites.

**Figure 4:**
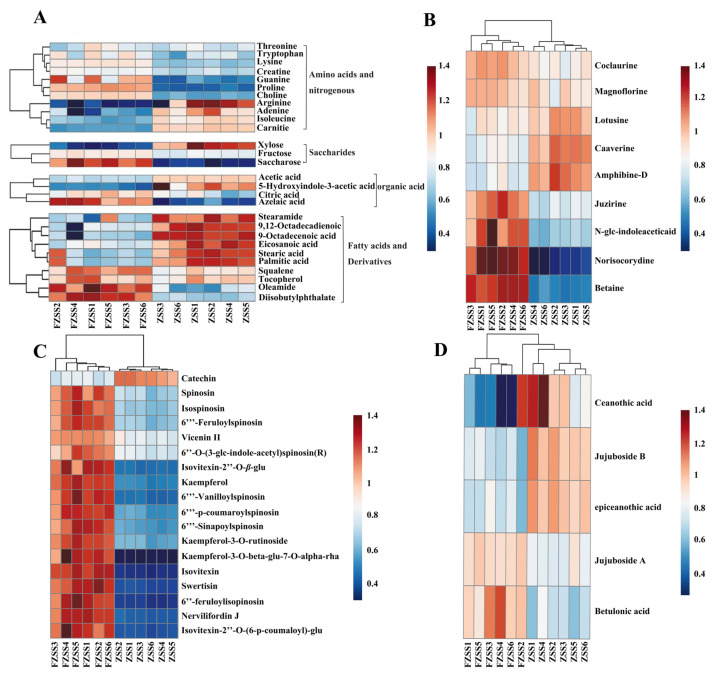
Heatmaps of differential metabolites between ZSS and FZSS. (**A**) Levels of 28 primary metabolites based on ^1^H NMR and GC–MS analyses (**B**) Levels of differential nitrogen-containing compounds; (**C**) Levels of differential flavonoids; (**D**) Levels of differential Saponins.

To provide a better overview of the content changes in differential compounds between ZSS and FZSS, a semi-quantitative analysis was performed using a hierarchical clustering heatmap. The heatmap demonstrated that the metabolites formed two distinct clusters (Figure 4A), indicating a significant difference in metabolite profiles between ZSS and FZSS. In the cluster gram, each variable is represented by a color-coded box: red indicates a higher level, and black indicates a lower level of the metabolite. As shown in Figure S4, the levels of specific metabolites were notably altered. The FZSS group contained higher amounts of lysine, proline, glutamine, creatine, choline, fructose, sucrose, acetic acid, acetoacetic acid, 6‴-feruloylspinosin, spinosin, and magnoflorine, but lower amounts of isoleucine, carnitine, and xylose, compared to the ZSS group.

The ^1^H NMR analysis of ZSS indicated that the levels of certain amino acids, nitrogen-containing compounds, sugars, and organic acids increased significantly (*p* < 0.05) after processing. As frying time increases, protein structures are disrupted, releasing a large number of free amino acids and leading to an increase in their content. In addition, the organic acid content in FZSS increased from 0.97% to 1.21% (Table S1). This rise in organic acids may be attributed to the Maillard reaction, which occurs between free amino groups and carbonyl groups of reducing sugars. The consumption of amino groups during this reaction can lead to a decrease in pH [34]. Furthermore, the observed increase in fructose is likely a result of polysaccharide degradation. It has been reported that polysaccharides in traditional Chinese medicines could degrade into oligosaccharides or monosaccharides during processing [31].

#### 3.3.2. GC–MS Data Analysis

Similarly, PCA and OPLS-DA were performed on the metabolome data acquired by GC–MS. The stability of the instrument and the suitability of the data pretreatment are demonstrated by the tight clustering of the QC samples in Figure 3C. The two-dimensional PCA plot explains 84.7% of the total variance, with the PC1 accounting for 72.4% and the PC2 for 12.3%. The ZSS samples were predominantly distributed on the positive axis of PC1, whereas the FZSS samples were clustered on the negative axis. The OPLS-DA model (Figure S3C) showed a clear separation between the ZSS and FZSS groups, suggesting a significant alteration in fatty acid composition after processing. The model demonstrated high goodness-of-fit and predictability (*R*^2^*X* = 0.999, *R*^2^*Y* = 0.991, *Q*^2^ = 0.982) (Figure S3D).

A total of 7 differential metabolites were identified as potential biomarkers for distinguishing between ZSS and FZSS. To compare the levels of fatty acid composition, a clustering heatmap and semi-quantitative analysis were employed. The heatmap (Figure 4A) revealed that the levels of most fatty acids were relatively higher in ZSS, whereas the levels of terpenoids (squalene) and vitamins (*γ*-tocopherol) were significantly increased in FZSS. Box plot analysis (Figure S5, Table S2) confirmed that the contents of stearic acid, palmitic acid, eicosanoic acid, 9-octadecenoic acid, and 9,12-octadecadienoic acid significantly decreased after processing (*p* < 0.05).

The lower proportion of unsaturated fatty acids after high-temperature is primarily attributed to the higher lipid oxidation rate [35], thus altering its carbon chain formation, which were generated olefin components (1,3,12-nonadecatriene), aldehyde components (2,4-decadienal, (E,E)-),9,17-octadecadienal,(Z)-,2-methyl-Z,Z-3,13-octadecadienol). The Saturated fatty acids (SFAs) content increased from 0.90% to 6.75%, while the UFAs content decreased from 79.44% to 48.02%, which is consistent with the results reported in the literature [18].

#### 3.3.3. LC-MS Data Analysis

PCA and OPLS-DA macroscopically indicated the obvious differences in secondary differential metabolites between ZSS and FZSS. The PCA score plots (Figure 3E,G) revealed a clear separation between the ZSS and FZSS groups in both positive and negative ion modes. In the negative ion mode, the first two principal components accounted for 79.5% of the total variance, while in the positive ion mode, they explained 79.1%. The QC samples clustered tightly near the origin in both modes, indicating excellent analytical stability. Furthermore, the OPLS-DA score plots (Figure S3E,G) demonstrated distinct clustering of the two groups. The established OPLS-DA models showed high goodness-of-fit and predictability (positive mode: *R*^2^*X* = 0.873, *R*^2^*Y* = 0.998, *Q*^2^ = 0.993; negative mode: *R*^2^*X* = 0.778, *R*^2^*Y* = 0.999, *Q*^2^ = 0.993). The reliability of these models was further confirmed by 200 permutation tests (Figure S3F,H), which showed no signs of overfitting. Collectively, these results provide a credible basis for the identification of differential metabolites.

A total of 43 differential metabolites were identified between ZSS and FZSS (Table S4), comprising 18 flavonoids, 9 nitrogen-containing compounds, 5 saponins, 3 fatty acids, and 8 other compounds. These metabolites serve as potential biomarkers for not only distinguishing ZSS from FZSS but also for elucidating the differences in their secondary metabolite profiles.

- Nitrogen-containing compounds;

Nitrogen-containing compounds were essential compounds in ZSS, which exert their sedative hypnotic effect through the Trp/5-HT/melatonin pathway. Nitrogen-containing compounds in ZSS had been confirmed to have significant anti-depressive activity [36]. A total of nine potential alkaloid biomarkers were identified between ZSS and FZSS (Table S4). These nitrogen-containing compounds primarily fall into two categories: cyclopeptide alkaloids and isoquinoline alkaloids. The heatmap analysis (Figure 4B) indicated that the overall levels of isoquinoline alkaloids were relatively higher in FZSS than in ZSS. Box plot analysis (Figure S6) further revealed that the relative contents of specific isoquinoline alkaloids—namely coclaurine, magnoflorine, juzirine, N-glc-indoleacetic acid and norisocorydine—were significantly increased after processing (*p* < 0.01). In contrast, the relative contents of other alkaloids, including the isoquinoline alkaloids caaverine and lotusine, as well as cyclopeptide alkaloids such as amphibine-D, were significantly decreased (*p* < 0.01).

- Flavonoids;

The flavonoids were primary component in ZSS, which accounted for 30.55% of the total number of components. Previous studies have shown that flavonoids in ZSS possess anxiolytic and sedative-hypnotic activities [14,37]. In this study, a total of 18 potential flavonoid biomarkers were tentatively identified by LC-MS, primarily classified into 14 flavonoid *C*-glycosides, 3 flavonoid *O*-glycosides, and 1 flavanol. Heatmap analysis (Figure 4C) indicated that flavonoid *C*-glycosides and *O*-glycosides were more abundant in FZSS, whereas flavanols catechin were more abundant in ZSS. Box plot analysis (Figure S6) confirmed that the levels of the *C*-glycosides (including spinosin, isospinosin, swertisin, 6‴-vanilloylspinosin, 6‴-sinapoylspinosin, 6‴-feruloylspinosin, 6‴-p-coumaroylspinosin, etc.) and the *O*-glycosides (kaempferol, kaempferol-3-O-rutinoside) were significantly higher in FZSS than in ZSS. In contrast, the content of catechin was significantly lower in FZSS. This decrease may originate from the decomposition and isomerization of epigallocatechin gallate (EGCG) during the baking process [38].

- Saponins;

A total of five potential saponin biomarkers were tentatively identified by LC-MS. Heatmap analysis (Figure 4D) indicated that most dammarane-type tetracyclic triterpenoids and lupane-type pentacyclic triterpenoids were predominantly present in FZSS, whereas ceanothane-type pentacyclic triterpenoids were more abundant in ZSS. Box plot analysis (Figure S6) confirmed that the levels of jujuboside A and betulinic acid were significantly increased in FZSS (*p* < 0.01). Conversely, the contents of jujuboside B, ceanothic acid, and epiceanothic acid were significantly higher in ZSS than in FZSS (*p* < 0.01).

- Fatty acid derivatives;

Since the samples for UHPLC-Q-Exactive Orbitrap-MS/MS analysis were defatted with petroleum ether prior to extraction, only three fatty acid derivatives—diisobutyl phthalate, oleamide, and stearamide—were detected in ZSS and FZSS. Heatmap analysis (Figure 4A) revealed that the levels of diisobutyl phthalate and oleamide were relatively higher in FZSS than in ZSS, whereas ZSS contained relatively higher levels of stearamide. Oleamide is a bioactive signaling molecule with similarities to endocannabinoids. It participates in sleep regulation through the CB1 receptor and demonstrates sleep-improving and antidepressant effects [39,40,41]. These results suggest that the calming efficacy of FZSS may be enhanced by its increased oleamide content.

- others;

Simultaneously, UPLC-MS analysis detected organic acids and nitrogenous compounds in ZSS and FZSS, including three organic acids and five amino acids/amino acid derivatives. Box plot analysis (Figure S6) revealed that the levels of arginine, tryptophan, citric acid, and azelaic acid decreased significantly in FZSS (*p* < 0.05). This decrease in citric acid is consistent with findings in roasted coffee, where thermal degradation leads to its reduction [42]. Conversely, the contents of guanine and adenine increased significantly in FZSS (*p* < 0.05).

### 3.4. Targeted Quantitative Analysis

#### 3.4.1. Quantitative Analysis by GC–MS

9-Octadecenoic acid and 9,12-octadecadienoic acid are the main components of UFAs, while stearic acid, palmitic acid, and eicosanoic acid are the essential components of SFAs in ZSS [19,38]. GC–MS-based multivariate statistical analysis identified these five fatty acids as potential biomarkers for distinguishing ZSS from FZSS. Additionally, γ-tocopherol and squalene were identified as differential metabolites. Consequently, these seven compounds were selected for targeted metabolomic analysis by GC–MS.

To quantify the content changes of these fatty acids following the processing of ZSS, an internal standard method was applied. All calibration curves exhibited excellent linearity (r > 0.999) across the tested concentration ranges (Table S5).

**Figure 5:**
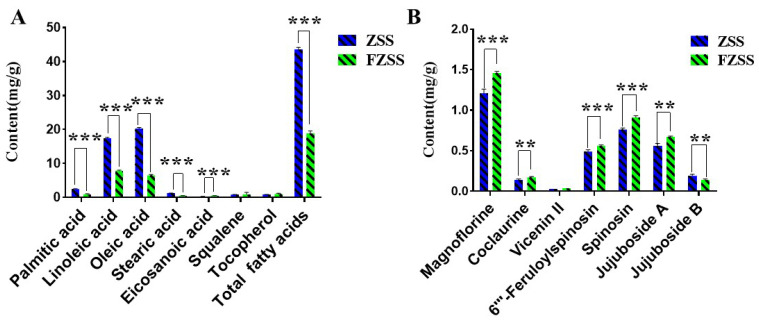
Targeted quantification of metabolites in ZSS and FZSS. (**A**) Fatty acid levels determined by GC–MS. (**B**) Secondary metabolite levels determined by HPLC-DAD-ELSD. Different letters above bars indicate significant differences (** *p* < 0.01, *** *p* < 0.001).

As shown in Figure 5A and Table S7, quantitative analysis revealed that the total fatty acid content in FZSS (18.94 ± 0.66 mg/g) was significantly lower than that in ZSS (43.68 ± 0.52 mg/g), representing a 56.6% decrease. The amounts of the main UFAs in ZSS, namely 9,12-octadecadienoic acid and (Z)-9-octadecenoic acid, dropped dramatically from 17.45 ± 0.22 mg/g and 20.33 ± 0.26 mg/g to 7.82 ± 0.21 mg/g and 6.51 ± 0.24 mg/g in FZSS, respectively. The contents of SFAs—palmitic acid, stearic acid, and eicosanoic acid—also decreased significantly in FZSS, by 64.22%, 64.06%, and 50.01%, respectively (*p* < 0.001). In contrast, the levels of γ-tocopherol and squalene showed no significant difference between ZSS and FZSS. These findings are consistent with the results from the GC–MS metabolomics analysis.

#### 3.4.2. Quantitative Analysis by HPLC-DAD-ELSD

Based on the framework of quality markers (Q-markers) and their fundamental principles [43], our previous study identified magnoflorine, jujuboside A, jujuboside B, 6‴-feruloylspinosin, spinosin, coclaurine, and vicenin II as potential biomarkers for distinguishing ZSS from FZSS [20]. Following the principle of testability, we quantitatively analyzed these seven Q-markers using HPLC-DAD-ELSD to explore differences in the content of major secondary metabolites between ZSS and FZSS.

As shown in Table S6, the calibration curves for all components exhibited good linearity within their respective concentration ranges (r > 0.999). The results in Figure 5B and Table S8 indicate that the contents of all six quantified components changed significantly after processing, except for vicenin II. The levels of both nitrogen-containing compounds increased: the content of magnoflorine rose from 1.21 ± 0.05 mg/g to 1.46 ± 0.02 mg/g (*p* < 0.001), and the content of coclaurine increased significantly from 0.14 ± 0.01 mg/g to 0.17 ± 0.01 mg/g (*p* < 0.01). The saponin contents showed divergent trends. The content of jujuboside A in FZSS (0.67 ± 0.01 mg/g) was significantly higher than that in ZSS (0.56 ± 0.03 mg/g), representing an increase of 19.64%. In contrast, the content of jujuboside B decreased significantly by 26.32% in FZSS compared to ZSS.

Targeted analysis results indicated that the contents of jujuboside A and spinosin increased after stir-frying. These two components have been proven to be the main components for the sedative effect of ZSS. Specifically, jujuboside A exerts its effects not only by adjusting γ-aminobutyric acid receptor subunit mRNA expression but also by down-regulating the secretion of relevant inflammatory cytokines in the intestinal mucosal system, thereby affecting the intercellular cytokine network of brain neurons [44]. Meanwhile, the effect of spinosin on rapid eye movement sleep in pentobarbital-treated rats may be related to postsynaptic 5-HT1A receptors [45]. These results confirm that the sedative effect of ZSS is enhanced after stir-frying.

In summary, the increased content of the five secondary metabolites in FZSS may be attributed to the facilitated release of active components during processing. In contrast, the content of jujuboside B was significantly reduced. Notably, bacopaside IV and jujubogenin were detected as unique components in FZSS. Based on literature evidence, bacopaside IV is likely formed by the hydrolysis of jujuboside B, involving the removal of one xylose (150.1) and one glucose (162.1) molecule. Subsequently, bacopaside IV can be further hydrolyzed by losing one rhamnose (164.16) and one arabinose (150.13) molecule to yield jujubogenin ([M-H]^−^ *m*/*z* 471.2). As shown in Figure S7, it has been conjectured that bacopaside IV and jujubogenin are a series of glycosyl hydrolysates of jujuboside B in the processing. The hydrolysis reaction of jujuboside B during high-temperature heating is similar to the biotransformation reactions reported in the literature for intestinal flora [46].

## 4. Conclusions

In this work, ^1^H NMR, GC–MS, and LC-MS analyses were successfully employed to characterize the chemical profile changes in ZSS before and after processing. The results indicated that the overall content of fatty acids decreased, while the levels of most primary metabolites (e.g., sugars, amino acids) and secondary metabolites (e.g., alkaloids, flavonoids) increased significantly after processing. Specifically, among the secondary metabolites, the levels of jujuboside A and betulinic acid increased markedly, whereas those of jujuboside B, ceanothic acid, and epiceanothic decreased. Targeted quantitative validation results for fourteen key fatty acid and secondary metabolite components were consistent with the untargeted metabolomics findings. This study enhances our understanding of the biochemical transformations occurring during the processing of ZSS. More importantly, it provides a comprehensive basis for quality control by employing an integrated untargeted and targeted metabolomics strategy. This approach offers a more systematic chemical profile compared to conventional methods reliant on limited markers as it captures global compositional changes and pinpoints critical components that both increase and decrease, thereby establishing a superior foundation for scientific processing parameters and robust quality control standards. In further studies, we will continue to explore the differences in pharmacological effects between raw ZSS and FZSS, so as to further to clarify the specific component groups responsible for its efficacy improvement.

## Data Availability

The original contributions presented in this study are included in the article/Supplementary Materials, and further inquiries can be directed to the corresponding authors.

## Supplementary Materials

The following supporting information can be downloaded at: https://www.mdpi.com/article/10.3390/foods14213771/s1, Figure S1: ^1^H-^1^H COSY spectra of ZSS in the *δ* 1.0–8.0 region; Figure S2: The base peak ion chromatograms (BPC) of ZSS and FZSS samples in negative mode using UHPLC-Q-Orbitrap-MS; Figure S3: Orthogonal partial least-squares discriminant analysis (OPLS-DA) models comparing ZSS and FZSS; Figure S4: Box plots comparing metabolite levels in ZSS and FZSS based on ^1^H NMR analysis; Figure S5: Box plots comparing metabolite levels in ZSS and FZSS based on GC–MS; Figure S6: Box plots comparing secondary metabolite levels in ZSS and FZSS based on UPLC-Q-Exactive Orbitrap; Figure S7: The possible and transformation mechanism of jujuboside B in processing. Table S1: ^1^H NMR assignments of major metabolites from ZSS and ZMS aqueous methanol extracts; Table S2: Detailed information of the 39 compounds identified or tentatively characterized by GC–MS from two seeds; Table S3: Detailed information of compounds identified or tentatively characterized from two seeds based on UHPLC-Q-Orbitrap-MS; Table S4: Differential metabolite expression (VIP > 1.00) in ZSS and FZSS based on UHPLC-Q-Orbitrap-MS; Table S5: Calibration curve data for quantitative analysis based on GC–MS; Table S6: Calibration curve data for quantitative analysis based on HPLC; Table S7: The content of seven fatty acid compositions; Table S8: The content of seven secondary metabolic components.

## Abbreviations

The following abbreviations are used in this manuscript: GC–MS, gas chromatography mass spectrometry; HCA, hierarchical clustering analysis; LC–MS, liquid chromatography mass spectrometry; NMR, nuclear magnetic resonance; OPLS-DA, orthogonal partial least squares-discriminant analysis; PCA, Principal component analysis; Q-markers, quality markers; SFAs, Saturated fatty acids; TIC, total ion chromatograms; UFAs, unsaturated fatty acids; VIP, variable importance in projection; ZSS, Ziziphi Spinosae Semen; FZSS, Fried Ziziphi Spinosae Semen.
