# Supplementary material for: Analysis of Components in Ziziphi Spinosae Semen Before and After Processing Based on Targeted and Untargeted Metabolomics
Source: Foods. 2025 Nov 3;14(21):3771. doi: 10.3390/foods14213771 (PMC12607712; doi:10.3390/foods14213771)
Supplement: Supplementary file 1 [file foods-14-03771-s001.zip › foods-3932303-supplementary.pdf]

Supplementary Materials for

Analysis of Components in Ziziphi Spinosae Semen Before and After Processing Based on Targeted and Untargeted Metabolomics

Ruiqi Yang [1†], Ze Li [1†], Lulu Dong [1], Yiran Heng [1], Lianglei Song [1], Lijun Guo [1], Xiangping Pei [1], Yan Yan [2*], Chenhui Du [1*]

1 School of Traditional Chinese Materia Medica, Shanxi University of Chinese Medicine, Taiyuan 030619, China; yangruiqi@sxtcm.edu.cn (R.Y.); 939948694@qq.com (Z.L.); 1612989310@qq.com (L.D.); hengyiran1@163.com (Y.H.); songlianglei2000@163.com (L.S.); guolijun@sxtcm.edu.cn (L.G.); peixp69@163.com (X.P.).

2 Modern Research Center for Traditional Chinese Medicine, Shanxi University, Taiyuan 030006, China.

* Correspondence: yanyan520@sxu.edu.cn (Y.Y.); dch@sxtcm.edu.cn (C.D.).

† These authors contributed equally to this work.

Figure S1

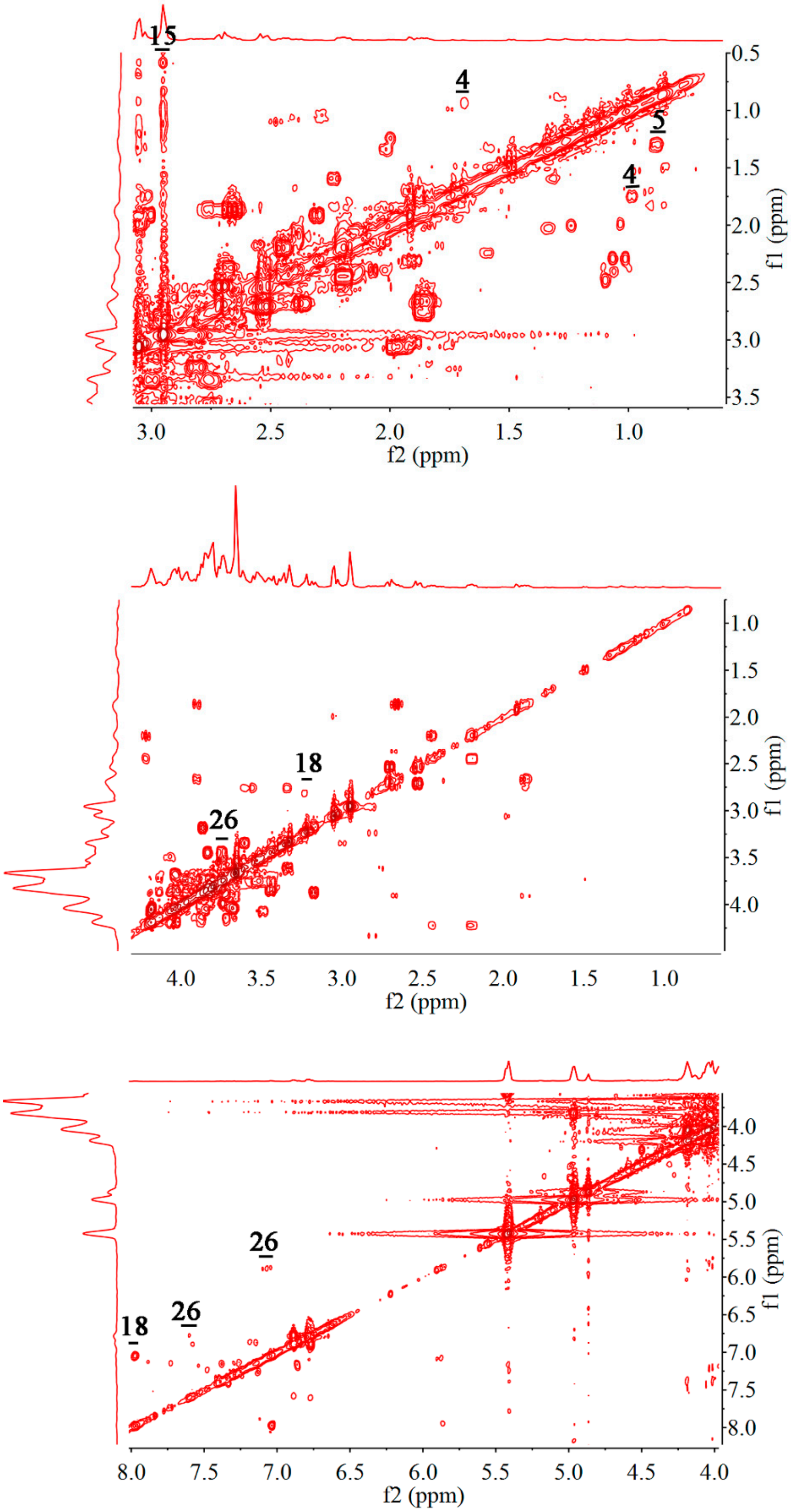

Figure S1 ^1H-^1H COSY spectra of ZSS in the δ 1.0-8.0 region. The observed signals are as follows: 15, magnoflorine; 18, spinosin; and 26, 6'''-feruloylspinosin.

Figure S2

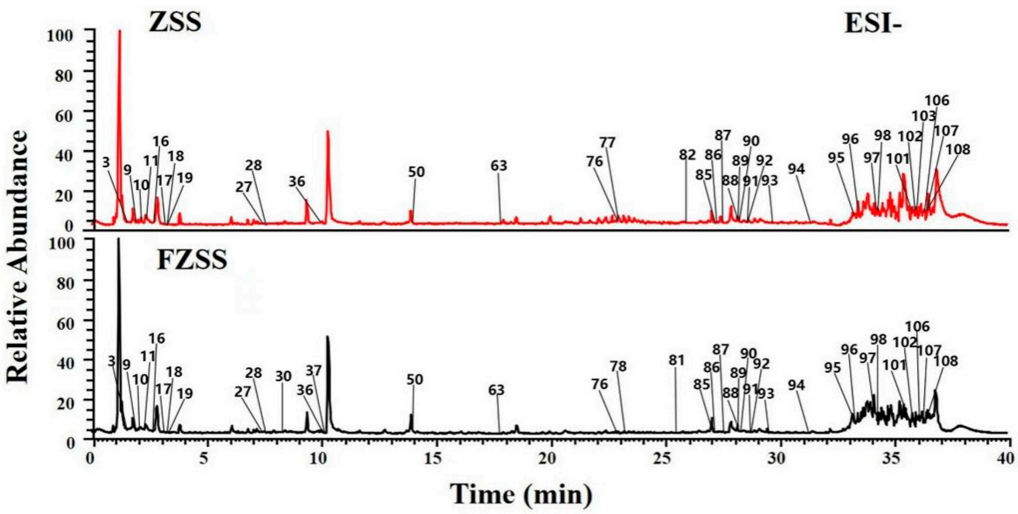

Figure S2 The base peak ion chromatograms (BPC) of ZSS and FZSS samples in negative mode using UHPLC-Q-Orbitrap-MS

Figure S3

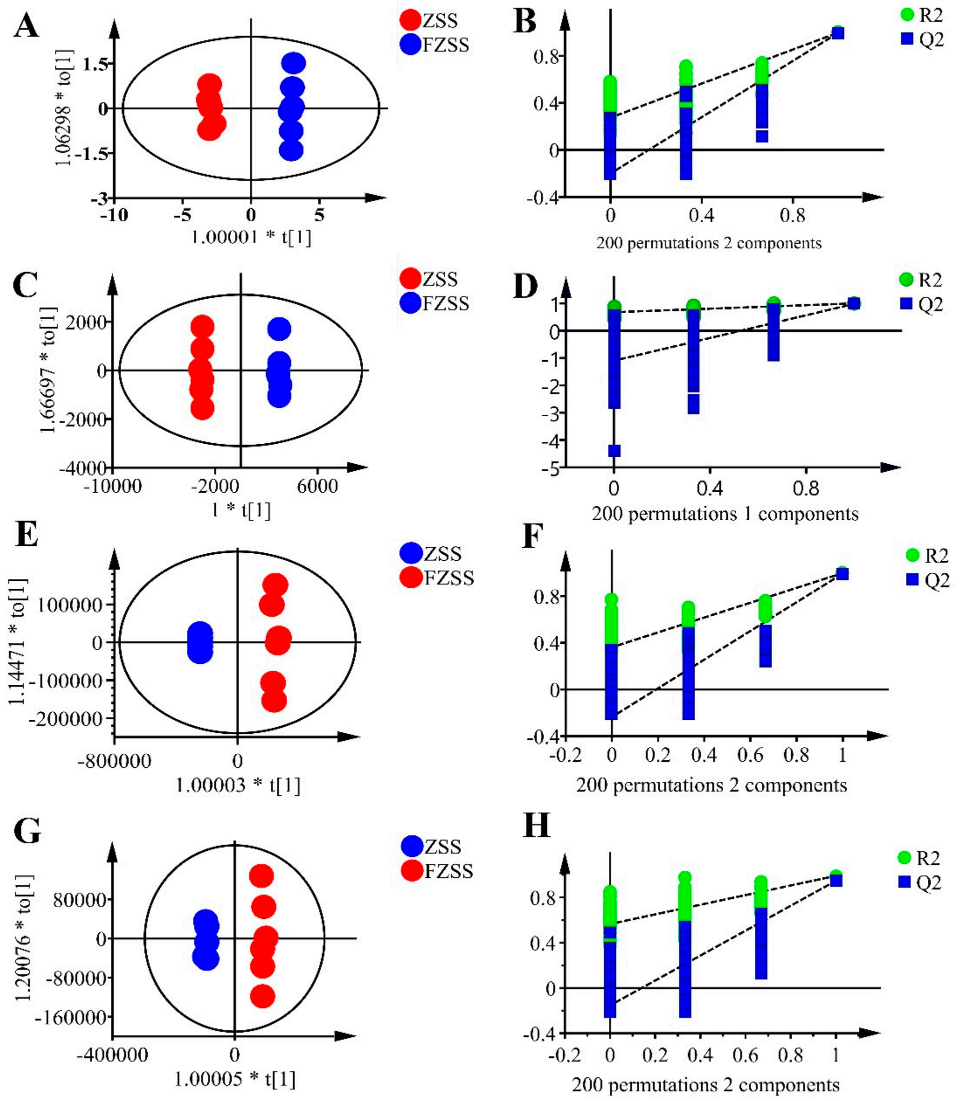

Figure S3. Orthogonal partial least-squares discriminant analysis (OPLS-DA) models comparing ZSS and FZSS. (A, B) Primary metabolites (^{1}H NMR): (A) score plot, (B) permutation test. (C, D) Fatty oils (GC-MS): (C) score plot, (D) permutation test; (E, F) Secondary metabolites, LC-MS positive mode: (E) score plot, (F) permutation test; (G, H) Secondary metabolites, LC-MS negative mode: (G) score plot, (H) permutation test.

Figure S4

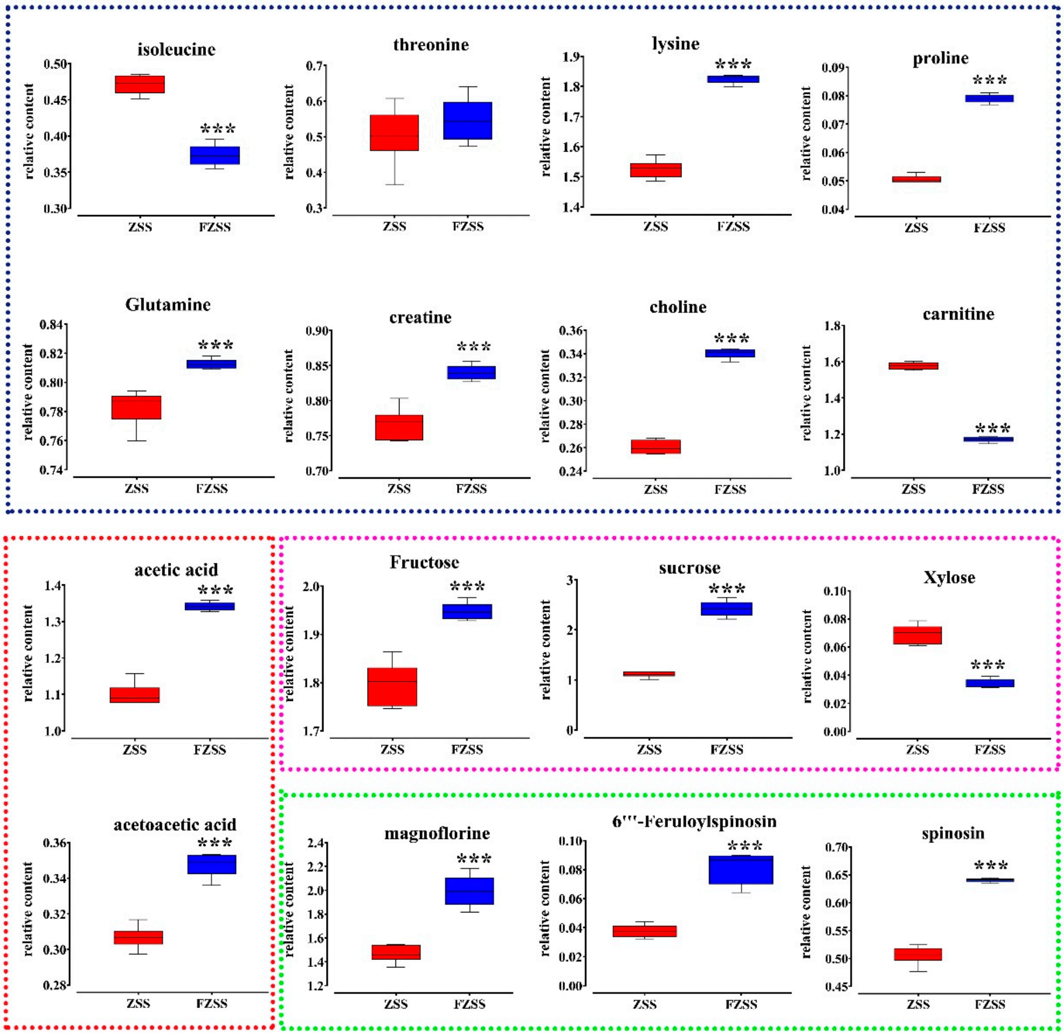

Figure S4. Box plots comparing metabolite levels in ZSS and FZSS based on ^{1}H NMR analysis. (significant differences, *$p < 0.05$, **$p < 0.01$, ***$p < 0.001$.)

Figure S5

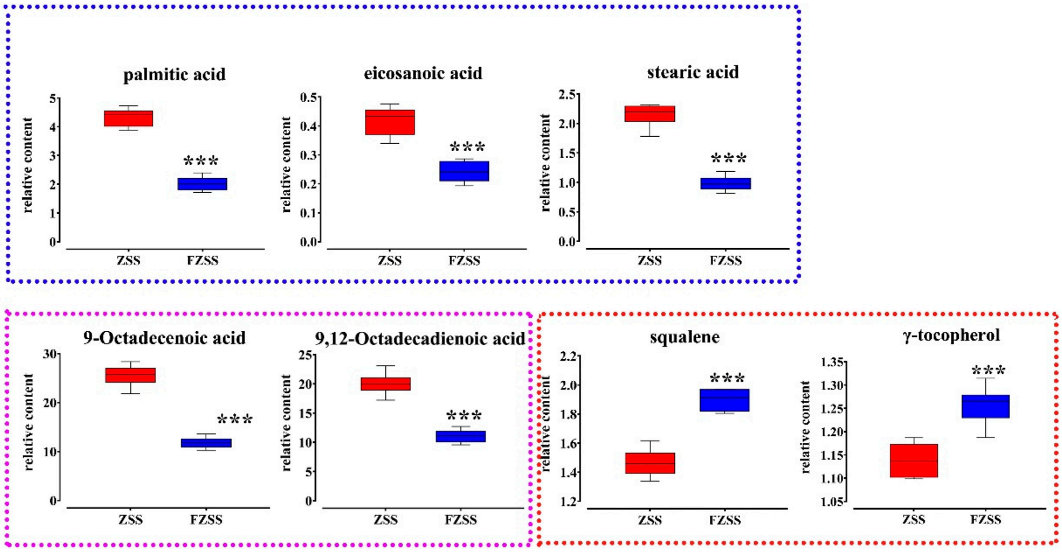

Figure S5. Box plots omparing metabolite levels in ZSS and FZSS based on GC-MS. (significant differences, *$p < 0.05$, **$p < 0.01$, ***$p < 0.001$.)

Figure S6

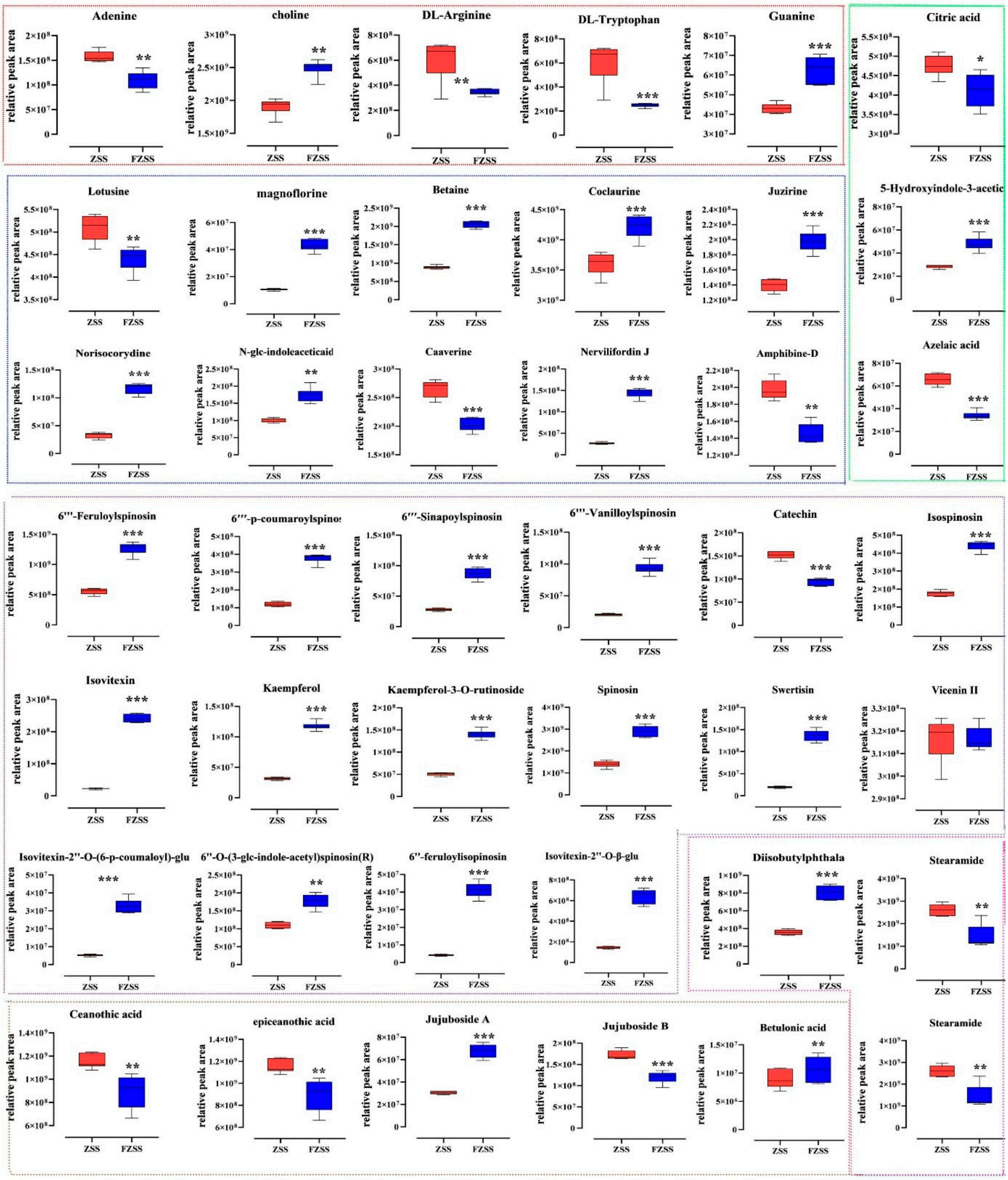

Figure S6. Box plots comparing secondary metabolite levels in ZSS and FZSS based on UPLC-Q-Exactive Orbitrap.
(significant differences, **p* < 0.05, ***p* < 0.01, ****p* < 0.001.)

Figure S7

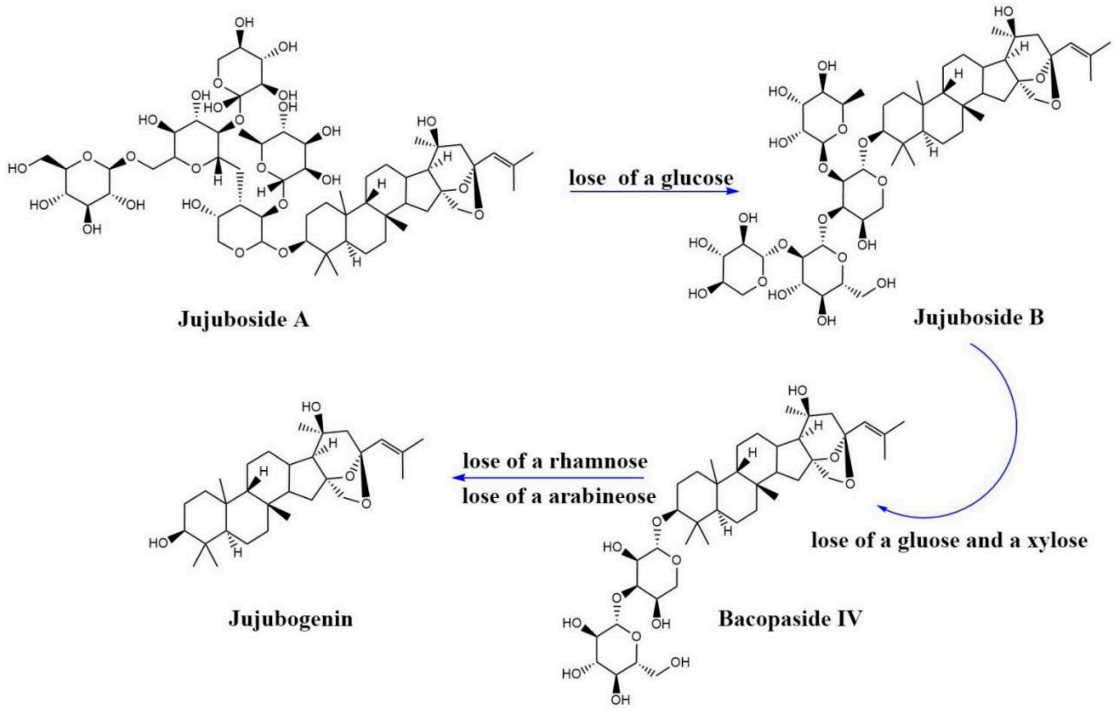

Figure S7. The possible and transformation mechanism of Jujuboside jujuboside B in processing.

Table S1

Table S1 ^{1}H NMR assignments of major metabolites from ZSS and ZMS aqueous methanol extracts

No.	Metabolites	δ_H	ZSS%	FZSS%
H1	Leucine	0.95 (t, 7.2,6.6)	0.08±0.006	0.08±0.005
H2	Isoleucine	1.00 (d, 6.6)	0.16±0.009	0.13±0.008
H3	Valine	1.03 (d, 6.6)	0.08±0.005	0.05±0.006
H4	Jujuboside A	1.07(d,7.2),1.18(s)	0.06±0.006	0.06±0.005
H5	Betulinic acid	1.26 (s)	0.15±0.003	0.14±0.004
H6	Threonine	1.33(d,6.6)	0.21±0.06	0.21±0.04
H7	Alanine	1.49 (d, 7.2)	0.15±0.01	0.14±0.01
H8	Lysine	1.89 (m)，3.74 (t, 5.4, 6)	0.13±0.003	0.15±0.002
H9	Acetic acid	1.92 (s)	0.97±0.03	1.19±0.03
H10	Proline	2.00 (m)，2.33 (m)	0.08±0.003	0.07±0.002
H11	*N*-acetylglutamate	2.02 (s)	0.09±0.002	0.09±0.004
H12	Glutamic acid	2.07 (m)	0.08±0.005	0.11±0.003
H13	Glutamine	2.13 (m), 2.44 (m)	0.09±0.006	0.08±0.003
H14	Glucuronic acid	2.24 (s)	0.04±0.002	0.05±0.003
H15	γ-aminobutyric acid	2.31 (t, 7.2, 7.2)	0.12±0.007	0.10±0.006
H16	Dimethylglycine	2.91 (s)	0.02±0.004	0.03±0.003
H17	Magnoflorine	2.95 (s)	1.34±0.08	1.95±0.13
H18	Creatine	3.02 (s), 3.93 (s)	0.15±0.01	0.21±0.03
H19	Creatinine	3.04 (s)	0.09±0.004	0.08±0.005
H20	Choline	3.19 (s)	0.20±0.007	0.28±0.005
H21	Carnitine	3.22 (s)	0.62±0.08	0.68±0.06
H22	Betaine	3.26 (s)	0.12±0.006	0.10±0.005
H23	6'''-Feruloylspinosin	3.33 (m), 4.59 (d,7.8)	1.25±0.03	1.41±0.03
H24	Ethylacetoacetate	3.46 (s)	0.31±0.01	0.35±0.01
H25	Fructose	3.62 (s)	1.20±0.04	1.25±0.04
H26	Saccharose	3.66 (s), 3.81 (m)	1.08±0.09	2.43±0.21
H27	Spinosin	3.95 (s), 4.00 (s)	0.30±0.02	0.26±0.01
H28	Xylose	5.20 (d, 3.6)	0.05±0.006	0.01±0.002
H29	α-glucose	5.23 (d, 3.60)	0.007±0.002	0.007±0.001
H30	Adenosine	6.03 (d, 6)	0.003±0.003	0.006±0.002
H31	Fumaric acid	6.53 (s)	0.004±0.002	0.008±0.001
H32	Tyrosine	6.90 (d, 7.8), 7.19 (d, 8.4)	0.04±0.003	0.05±0.002
H33	Histidine	7.07 (s)	0.02±0.003	0.03±0.002
H34	Phenylalanine	7.33 (m)	0.03±0.001	0.03±0.002
H35	Formic acid	8.44 (s)	6.93E±0.002	0.003±0.001
	organic acid content		0.97±0.03	1.20±0.03

s: single, d: dou.blet, t: triplet, m: multiple, dd: doublet of doublet.

Table S2

Table S2 Detailed information of the 39 compounds identified or tentatively characterized by GC-MS from two seeds

No.	Compound	Rt(min)	CAS	M	Identification method	Matched-		Relative amount		Type of compounds
						ZSS	FZSS	ZSS%	FZSS%	
G1	2,4-Decadienal, (E,E)-	3.242	25152-84-55	152.23	NIST 14	94	94	0.167±0.00	0.410±0.00	Fatty acid derivative
G2	Nonanoic acid (C9:0)	3.459	112-05-0	158.24	NIST 14	91	ND	0.0455±0.00	ND	Fatty acid
G3	Dimethyl phthalate	4.592	131-11-3	194.18	NIST 14	94	95	0.0511±0.00	0.151±0.00	Fatty acid derivative
G4	Myristic acid（C14:0)	10.434	544-63-8	228.37	NIST 14	97	ND	0.0553±0.00	ND	Fatty acid
G5	Pentadecanoic acid（C15:0)	12.185	1002-84-2	242.44	NIST 14	96	ND	0.0376±0.00	ND	Fatty acid
G6	Dibutyl phthalate	12.517	84-74-2	278.34	NIST 14	94	96	0.251±0.00	0.0524±0.00	Fatty acid derivative
G7	1,2-Benzenedicarboxylic acid, butyl 2-methylpropyl ester	12.523	103048-19-7	418.61	NIST 14	95	ND	0.130±0.00	ND	Fatty acid derivative
G8	Hexadecanoic acid, ethyl ester	13.015	628-97-7	284.48	NIST 14	91	94	0.0305±0.00	0.0728±0.00	Fatty acid derivative
G9	Palmitic Acid（C16:0)	13.976	57-10-3	256.42	NIST 14	99	99	1.156±0.01	2.261±0.01	Fatty acid
G10	12,15-Octadecadienoic acid, methyl	14.817	57156-97-5	294.47	NIST 14	94	ND	0.0505±0.00	ND	Fatty acid derivative
G11	2-Chloroethyl linoleate	14.817	25525-76-2	342.94	NIST 14	90	ND	0.0575±0.00	ND	Fatty acid derivative
G12	9,15-Octadecadienoic acid, methyl ester, (Z,Z)-	14.823	5489-59-8	294.47	NIST 14	97	ND	0.0612±0.00	ND	Fatty acid derivative
G13	trans-13-Octadecenoic acid, methyl	14.931	42199-38-2	296.49	NIST 14	91	ND	0.0561±0.00	ND	Fatty acid derivative
G14	9,17-Octadecadienal, (Z)-	14.977	56554-35-9	264.45	NIST 14	90	95	0.0115±0.00	0.139±0.00	Fatty acid derivative
G15	Linoelaidic acid（C18:2)	15.504	506-21-8	280.45	NIST 14	95	93	0.465±0.00	0.208±0.00	Fatty acid
G16	11-Dodecen-1-ol	15.607	35289-31-7	184.32	NIST 14	96	ND	0.0258±0.00	ND	Fatty acid derivative
G17	Heptadecanoic acid（C17:0)	15.881	506-12-7	270.45	NIST 14	90	95	0.0897±0.00	0.321±0.00	Fatty acid

Table S2 Detailed information of the 39 compounds identified or tentatively characterized by GC-MS from two seeds (continued)

No.	Compound	Rt(min)	CAS 号	M	Identification method	Matched-degree		Relative amount		Type of compounds
						ZSS	FZSS	ZSS%	FZSS%	
G18	9,12-Octadecadienoic acid, ethyl ester	16.288	544-35-4	308.50	NIST 14	99	99	0.191±0.00	0.513±0.00	Fatty acid derivative
G19	(E)-9-Octadecenoic acid ethyl ester	16.362	6114-18-7	310.51	NIST 14	99	99	0.247±0.00	0.590±0.00	Fatty acid derivative
G20	9,12-Octadecadienoic acid (Z,Z)-	17.581	544-35-4	280.45	NIST 14	95	99	2.828±0.20	3.601±0.15	Fatty acid
G21	9-Octadecenoic acid, (Z)-（C18:1)	17.724	103-23-1	282.46	NIST 14	99	ND	4.588±3.90	5.1±3.56	Fatty acid
G22	Stearic acid（C18:0)	18.445	57-11-4	284.48	NIST 14	99	99	1.568±0.00	0.0208±0.00	Fatty acid
G23	11-Eicosenoic acid, (Z)-（C20:1)	22.994	2462-94-4	310.51	NIST 14	99	99	1.021±0.00	0.1389±0.00	Fatty acid
G24	Hexanedioic acid, bis(2-ethylhexyl)	23.658	103-23-1	370.57	NIST 14	90	95	0.169±0.00	0.438±0.00	Fatty acid derivative
G25	Eicosanoic acid（C20:0)	23.658	506-30-9	312.53	NIST 14	95	99	0.296±0.00	0.504±0.00	Fatty acid
G26	Campesterol	27.24	474-62-4	400.69	NIST 14	N	95	ND	0.213±0.00	Steroid
G27	Behenic acid（C22:0)	27.56	112-85-6	340.58	NIST 14	95	99	0.298±0.00	0.669±0.00	Fatty acid
G28	Methyl 9,12-heptadecadienoate	27.886	6422-86-2	294.47	NIST 14	91	92	0.0759±0.00	0.0611±0.00	Fatty acid derivative
G29	Sucrose	28.43	57-50-1	342.30	NIST 14	N	93	ND	2.641±0.00	Saccharides
G30	Stigmasterol	28.619	83-48-7	412.70	NIST 14	98	99	0.497±0.00	1.643±0.00	Steroid
G31	1,3-Benzenedicarboxylic acid, bis(2-ethylhexyl) ester	29.059	137-89-3	390.56	NIST 14	91	91	0.607±0.00	1.112±0.00	Fatty acid derivative
G32	1,4-Benzenedicarboxylic acid, bis(2-ethylhexyl) ester	29.059	6422-86-2	390.56	NIST 14	91	ND	0.941±0.00	1.99±0.00	Fatty acid derivative
G33	Squalene	30.347	7683-64-9	410.72	NIST 14	99	99	1.221±0.00	4.655±0.00	Terpene
G34	Lignoceric acid（C24:0)	30.558	557-59-5	368.64	NIST 14	91	96	0.306±0.00	0.974±0.00	Fatty acid
G35	beta.-Sitosterol	31.194	83-46-5	414.72	NIST 14	99	99	3.434±0.56	7.728±0.04	Steroid
G36	Lup-20(29)-en-28-al, 3-(trimethylsilyl)oxy, (3.beta.)-	31.417	13159-28-9	440.71	NIST 14	93	99	1.470±0.01	5.736±0.01	Terpene

Table S2 Detailed information of the 39 compounds identified or tentatively characterized by GC-MS from two seeds (continued)

No.	Compound	Rt(min)	CAS 号	M	Identification method	Matched-degree		Relative amount		Type of compounds
						ZSS	FZSS	ZSS%	FZSS%	
G37	2-Methyl-Z,Z-3,13-octadecadienol	33.036	54-28-4	280.27	NIST 14	94	91	0.0596±0.00	0.394±0.00	Fatty acid derivative
G38	gamma.-Tocopherol	33.969	54-28-4	416.68	NIST 14	99	99	0.971±0.00	3.178±0.00	Vitamin
G39	1,3,12-Nonadecatriene	34.341	646052-22-4	262.47	NIST 14	95	91	0.0375±0.00	0.360±0.00	Fatty acid derivative
Saturated fatty acid								0.9%	6.75%	
Unsaturated fatty acid								79.44%	48.02%	
Fatty acid								5.85%	6.75%	
Fatty acid derivative								3.62%	6.17%	
terpene								3.93%	9.58%.	

Table S3

Table S3 Detailed information of compounds identified or tentatively characterized from two seeds based on UHPLC-Q-Orbitrap-MS

NO	Compound	RT[min]	Formula	weight	$[M+H]^+$	Adduc	Error(p	MS/MS	source	
1	Pipecolic acid	1.467	$C_6 H_{11} N$	129.078	130.086	[M+H]	0.78	130.0863,84.0808,82.0651,	ZSS	FZSS
2	Arginine	1.498	$C_6 H_{14}$	174.111	175.119	[M+H]	0.58	175.11925,158.09276,116.07107,70.06609	ZSS	FZSS
3	Sucrose	1.5	$C_{12} H_{22}$	342.116	341.108	$[M-H]^-$	-0.29	179.05490,143.03358,161.04456,119.03348,113.0228	ZSS	FZSS
4	α-Eleostearic acid	1.562	$C_4 H_8 N_2$	132.053	133.061	[M+H]	0.76	279.23187,261.22131,243.21152,209.15282,195.1379	ZSS	FZSS
5	Choline	1.566	$C_5 H_{13} N$	103.099	104.107	[M+H]	0.11	104.10749,86.09698,60.08174,58.06607	ZSS	FZSS
6	5-Hydroxymethyl-2-	1.665	$C_6 H_6 O_3$	126.031	127.039	[M+H]	0.80	127.03934,109.02897,81.03433,69.03444	ZSS	FZSS
7	Proline*	1.672	$C_5 H_9 N$	115.063	116.071	[M+H]	0.88	116.07101,70.06604,56.05040,98.06061	ZSS	ND
8	4-Guanidinobutyric	1.778	$C_5 H_{11} N_3$	145.085	146.092	[M+H]	0.69	146.09267,128.08209,111.05581,104.07115,,87.0448	ZSS	FZSS
9	4-Oxoproline	1.912	$C_5 H_7 N$	129.042	128.033	$[M-H]^-$	-0.78	128.03392,82.02809,84.04375,	ZSS	FZSS
10	Citric acid	2.057	$C_6 H_8 O_7$	192.027	191.019	$[M-H]^-$	-0.52	191.01892,173.00810,147.02863,111.00723	ZSS	FZSS
11	N-Acetyl-L-glutamate	2.222	$C_7 H_{11} N$	189.063	188.055	$[M-H]^-$	-0.53	188.05551,170.04491,146.04471,144.06534,128.0339	ZSS	FZSS
12	5-Aminovaleric acid	2.265	$C_5 H_{11} N$	117.078	118.086	[M+H]	0.86	118.08664,72.08171,,58.06610,55.05523	ZSS	FZSS
13	Uracil	2.298	$C_4 H_4 N_2$	112.027	113.034	[M+H]	0.90	113.03510,96.00856,70.02963,	ND	FZSS
14	Guanine	2.476	$C_5 H_5 N_5$	151.049	152.056	[M+H]	0.67	152.05682,135.03024,110.03532,109.05128	ZSS	FZSS
15	Adenine	2.545	$C_5 H_5 N_5$	135.054<br>4	136.062<br>134.045	[M+H]<br>$[M-H]^-$	0.75<br>-0.75	136.06195，119.03550，94.04096<br>134.04575,107.03468,92.02374,	ZSS	FZSS
16	Itaconic acid	2.611	$C_5 H_6 O_4$	130.026	129.017	$[M-H]^-$	-0.78	129.01793,86.03126,85.02784	ZSS	FZSS
17	Citraconic acid	3.101	$C_5 H_6 O_4$	130.026	129.017	$[M-H]^-$	-0.78	129.01793,85.02784	ZSS	FZSS
18	Xanthosine	3.202	$C_{10} H_{12}$	284.075	283.068	$[M-H]^-$	-0.35	283.06857,151.02499	ZSS	FZSS
19	Gallic acid	3.25	$C_7 H_6 O_5$	170.021	169.013	$[M-H]^-$	-0.59	169.01328,125.02302，107.01241，	ZSS	FZSS
20	Betaine	3.54	C5 H11	117.079	118.086	[M+H]	0.86	118.08659,58.06609,59.07391	ZSS	FZSS
21	3R-N-glc-3-hydroxy-<br>indoleacetc acid	4.26	$C_{16}H_{19}N$<br>O_9	369.105<br>9	370.113<br>368.098	[M+H]<br>$[M-H]^-$	0.27<br>-0.27	146.06029,190.05011,208.06070,212.07104,272.8886<br>144.04417,146.02344,158.05997,176.07062,368.0993	ND	FZSS

Table S3 Detailed information of compounds identified or tentatively characterized from two seeds based on UHPLC-Q-Orbitrap-MS (continued)

NO	Compound	RT[min]	Formula	weight	$[M+H]^+$	Adduc	Error(p	MS/MS	source	
22	Tryptophan*	6.517	$C_{11}H_{12}$	204.089	205.097	[M+H]	0.49	205.09723,159.09198,144.08101,142.06544,74.02441	ZSS	FZSS
23	Indoleacrylic acid	6.532	$C_{11}H_9N$	187.063	188.070	[M+H]	0.54	188.07085,170.06023,142.06538	ZSS	FZSS
24	5-Hydroxyindole-3-	6.787	$C_{10}H_9N$	191.058	192.065	[M+H]	0.53	192.06557,174.05,146.06029,	ZSS	FZSS
25	6-glc-coclaurine	6.81	$C_{16}H_{19}N$	353.111	354.118	[M+H]	0.28	286.14395,269.11737,237.09143,209.09634,175.0756	ZSS	FZSS
26	N-glc-indoleaceticaid	7.25	$C_{23}H_{29}N$	447.189	448.196	[M+H]	0.23	270.07632,234.07632,188.07050,174.05528,146.0603	ZSS	FZSS
27	4-Hydroxybenzoic acid	7.374	$C_7H_6O_3$	138.031	137.023	$[M-H]^-$	-0.73	137.02307,119.01243，93.03296，	ZSS	FZSS
28	Terephthalic acid	7.505	$C_8H_6O_4$	166.026	165.019	$[M-H]^-$	-0.61	165.01816,121.02807	ZSS	FZSS
29	Indole-3-acrylic acid	7.625	$C_{11}H_9N$	187.063	188.070	[M+H]	0.54	188.07079,170.06010,115.05463	ZSS	FZSS
30	3-Hydroxyanthranilic	8.291	C_7H_7N	153.042	152.034	$[M-H]^-$	-0.66	152.03413，108.04422	ND	FZSS
31	Magnocurarine	8.42	$C_{19}H_{24}N$	314.175	314.138	$[M]^+$	-0.01	269.11749,237.09105,175.07568,107.04971,58.06616	ZSS	FZSS
32	Coclaurine*	9.48	$C_{17}H_{19}N$	285.136	286.144	[M+H]	0.35	107.04965,269.11728,286.14389,175.07564,237.0911	ZSS	FZSS
33	Juzirine	9.89	$C_{17}H_{15}N$	281.105	282.112	[M+H]	0.36	282.11246,188.07068,156.04451,128.04968,283.1158	ZSS	FZSS
34	Vicenin II*	10.00	$C_{27}H_{30}O_1$<br>$_5$	594.158<br>4	595.165<br>593.130	[M+H]<br>$[M-H]^-$	0.17<br>-0.17	325.07056,457.11331,379.08118,295.06006,427.1029<br>593.15240,353.06711,297.07706,383.07779,413.0884	ZSS	FZSS
35	Norisocorydine	10.01	$C_{19}H_{21}N$	327.147	328.154	[M+H]	0.31	328.15439,283.09674,251.07138,265.08615,209.0967	ZSS	FZSS
36	Hovetrichoside C	10.01	$C_{21}H_{22}O_1$	450.116	449.108	[M-H]-	-0.22	125.02301,259.06146,151.00267,178.99760,215.0709	ZSS	FZSS
37	6-Hydroxycaproic acid	10.122	$C_6H_{12}O_3$	132.078	131.069	$[M-H]^-$	-0.76	131.07007,85.06429	ND	FZSS
38	Lotusine	11.83	$C_{19}H_{24}N$	314.175	314.175	[M+H]	0.00	269.11757,237.09076,209.09601,175.07607,143.0494	ZSS	FZSS
39	6'''-(4''''-O-glc)-<br>vanilloylspinosin	12.28	$C_{42}H_{48}O_2$<br>$_3$	920.258<br>6	921.265<br>919.251	[M+H]<br>$[M-H]^-$	0.11<br>-0.11	327.08658,151.03929,351.08682,297.07602,429.1186<br>757.19940,209.04506,167.03395,292.03746,307.0621	ZSS	FZSS
40	Vitexin*	12.80	$C_{21}H_{20}O_1$	432.105	433.112	[M+H]	0.23	283.06015,313.07071,337.07086,367.08151,397.0915	ZSS	FZSS
41	Isovitexin-2''-O-β-<br>glucopyranoside	12.98	C27H30<br>O15	594.158<br>4	595.165<br>593.151	[M+H]<br>$[M-H]^-$	0.17<br>-0.17	313.07065,433.11310,283.06003,337.07056,415.0625<br>293.04599,59.01227,413.08844,593.15234,71.01221	ZSS	FZSS

Table S3 Detailed information of compounds identified or tentatively characterized from two seeds based on UHPLC-Q-Orbitrap-MS (continued)

NO	Compound	RT[min]	Formula	weight	$[M+H]^{+}$	Adduc	Error(p	MS/MS	source	
42	Kaempferol 3-O-beta-glucopyranoside-7-O-	13.227	$C_{27}H_{30}O_{15}$	594.1584	595.166<br>593.151	[M+H]<br>$[M-H]^{-}$	0.17<br>-0.17	595.16577,433.11310,,415,10257,283.06006，<br>593.15259,255.02979,285.04062	ZSS	FZSS
43	Magnoflorine*	13.26	$C_{20}H_{24}N$	342.170	342.170	[M+H]	0.00	342.17017,58.06614,297.11227,265.08227,237.09106	ZSS	FZSS
44	6'''-(4''''-(O-glc)-feruloylspinosin	13.41	$C_{44}H_{50}O_{23}$	946.2742	947.281<br>945.267	[M+H]<br>$[M-H]^{-}$	0.11<br>-0.11	177.05473,327.08636,145.02864,351.08630,297.0759<br>783.21521,427.10254,292.03812,307.06189,235.0609	ZSS	FZSS
45	Camelliaside B	13.43	$C_{32}H_{38}O_{19}$	726.2007	727.208<br>725.193	[M+H]<br>$[M-H]^{-}$	0.14<br>-0.14	287.05502,85.02917,71.05007,97.02908,129.05468，<br>284.03268,255.02966,227.03436,725.194446,151.002	ZSS	FZSS
46	Isospinosin*	13.69	$C_{28}H_{32}O_{15}$	608.1741	609.182<br>607.167	[M+H]<br>$[M-H]^{-}$	0.17<br>-0.17	447.129096,285.07605,327.08649,85.02924,297.0760<br>292.03812,309.04153,281.04568,427.10446,117.0329	ZSS	FZSS
47	Caaverine	13.78	$C_{17}H_{17}N$	267.125	268.133	[M+H]	0.38	251.10654,219.08049,191.08557,237.08638,268.1330	ZSS	FZSS
48	Kaempferol	13.829	$C_{15}H_{10}$	286.047	287.055	[M+H]	0.35	287.05499,153.01855,213.05504,231.06480	ZSS	FZSS
49	Spinosin*	13.86	$C_{28}H_{32}O_{15}$	608.1741	609.181<br>607.166	[M+H]<br>$[M-H]^{-}$	0.17<br>-0.17	327.08643,297.07590,351.08643,429.11823,381.0969<br>292.03812,427.10306,307.06146,281.04514,59.01218	ZSS	FZSS
50	Rutin*	13.918	$C_{27}H_{30}$	610.153	609.147	$[M-H]^{-}$	-0.16	609.14563，301.03326，	ZSS	FZSS
51	Dibenzylamine	14.094	$C_{14}H_{15}$	197.120	198.128	[M+H]	0.51	198.12807,106.06563,65.03970,91.05494,	ZSS	FZSS
52	Isovitexin*	14.20	$C_{21}H_{20}O_{10}$	432.1056	433.113<br>431.098	[M+H]<br>$[M-H]^{-}$	0.23<br>-0.23	283.06018,295.06082,313.07089,337.07092,397.0610<br>311.05634,283.06122,341.06686,413.09842,323.0573	ND<br>ZSS	FZSS
53	Vitexin*	14.277	$C_{21}H_{20}$	432.105	433.113	[M+H]	0.23	433.11295,397.09198，337.07077，313.07086，	ZSS	FZSS
54	Swertisin*	14.88	$C_{22}H_{22}O_{10}$	446.1212	447.128<br>445.114	[M+H]<br>$[M-H]^{-}$	0.23<br>-0.23	297.07565,327.08633,351.08624,411.107115,267.065<br>297.04062,282.05338,328.83264,230.85469,310.8217	ZSS	FZSS
55	6'''-Pyridyloylspinosin	14.88	$C_{34}H_{35}N$	713.195	714.203	[M+H]	0.14	429.11917,351.08609,327.08340,297.07623,124.0397	ND	FZSS
56	Isoswertisin	15.55	$C_{22}H_{22}O_{1}$	446.121	447.128	[M+H]	0.23	447.12885,299.05581,327.08633,285.07578,313.0313	ZSS	FZSS
57	6'''-Vanilloylspinosin	16.02	$C_{36}H_{38}O_{18}$	758.2058	759.214<br>757.199	[M+H]<br>$[M-H]^{-}$	0.13<br>-0.13	327.08646,151.03925,351.08667,297.07623,429.1181<br>209.04443,167.03380,427.10211,757.196672,292.037	ZSS	FZSS

Table S3 Detailed information of compounds identified or tentatively characterized from two seeds based on UHPLC-Q-Orbitrap-MS (continued)

NO	Compound	RT[min]	Formula	weight	$[M+H]^+$	Adduc	Error(p	MS/MS	source	
58	Kaempferol-3-O-rutinoside*	16.06	$C_{27}H_{30}O_{15}$	594.1584	595.166<br>593.151	[M+H]<br>$[M-H]^-$	0.17<br>-0.17	287.05524,85.02922,71.05011,129.05493,57.03458<br>593.15265，285.04053,255.02985,227.03471	ZSS	FZSS
59	6'''-p-Hydroxylbenzoylspino	16.45	$C_{35}H_{36}O_{17}$	728.1952	729.203<br>727.188	[M+H]<br>$[M-H]^-$	0.14<br>-0.14	327.08633,121.02872,351.08640,297.07590,429.1182<br>137.02303,179.03400,93.03291,239.05582,427.10333	ZSS	FZSS
60	Isovitexin-2''-O-(6-p-coumaloyl)-	16.70	$C_{36}H_{36}O_{17}$	740.1952	741.203<br>739.188	[M+H]<br>$[M-H]^-$	0.14<br>-0.14	147.04427,313.07080,283.06018,337.07071,433.1128<br>293.04578,413.08871,161.05943,593.15582,205.0500	ND<br>ZSS	FZSS
61	Isovitexin-2''-O-(6-feruloyl)-	16.85	$C_{37}H_{38}O_{18}$	770.2058	771.213<br>769.199	[M+H]<br>$[M-H]^-$	0.13<br>-0.13	177.05475,313.07062,145.02859,283.06006,337.0708<br>293.04581,413.08850,235.06062,193.04002,134.0360	ZSS	FZSS
62	Nervilifordin J	16.85	$C_{37}H_{38}O_{1}$	770.205	771.214	[M+H]	0.13	433.11362,415.10318,379.08170,367.08154,337.0713	ZSS	FZSS
63	Azelaic acid	17.736	C_{9} H_{16}	188.104	187.096	$[M-H]^-$	-0.54	187.09669,125.09565，123.08043，	ZSS	FZSS
64	Asimilobine	17.84	$C_{17}H_{17}N$	267.125	268.104	[M+H]	0.37	251.10686,219.08078,191.08583,237.0852,236.08322	ZSS	FZSS
65	6'''-Sinapoylspinosin	17.94	$C_{39}H_{42}O_{19}$	814.2320	815.240<br>813.225	[M+H]<br>$[M-H]^-$	0.12<br>-0.12	207.06548,327.08655,175.03923,351.08380,297.0759<br>427.10400,292.03821,190.02623,307.06235,265.0719	ZSS	FZSS
66	6'''-Dihydrophaseoylspino	17.95	$C_{43}H_{52}O_{19}$	872.3102	873.317<br>871.303	[M+H]<br>$[M-H]^-$	0.12<br>-0.12	327.08640,351.08636,297.07593,323.09155<br>427.10324,307.06128,607.16168,292.03809,309.0417	ZSS	FZSS
67	6'''-p-coumaroylspinosin	18.29	$C_{37}H_{38}O_{17}$	754.2108	755.218<br>753.203	[M+H]<br>$[M-H]^-$	0.13<br>-0.13	147.04428,327.08643,351.08667,297.07578,429.1169<br>145.02815,753.20410,163.03888,292.03802,119.0486	ZSS	FZSS
68	6'''-Feruloylspinosin*	18.48	$C_{38}H_{40}O_{18}$	784.2214	785.228<br>783.215	[M+H]<br>$[M-H]^-$	0.13<br>-0.13	177.05452,327.08597,145.02843,351.08600,297.0755<br>783.216674,134.03598,292.03796,427.10394,193.049	ZSS	FZSS
69	6''-O-(3-glc-indole-	19.67	$C_{44}H_{49}N$	943.274	944.283	[M+H]	0.11	764.21826,602.17010,489.13788,411.10861,393.0969	ZSS	FZSS
70	6''-O-(3-glc-indole-	20.00	$C_{44}H_{49}N$	943.274	944.282	[M+H]	0.11	602.16559,411.10687,393.09711,351.08655,327.0865	ZSS	FZSS
71	Mucronine J	20.39	$C_{27}H_{40}N_{4}$	484.304	485.312	[M+H]	0.21	114.12814,72.08164,485.31241,58.06610,115.13145	ZSS	FZSS
72	Nornuciferine	20.61	$C_{18}H_{19}N$	281.141	282.112	[M+H]	0.35	265.12228,250.09883,234.10339,266.12585,282.1490	ZSS	FZSS
73	6''-O-(3-glc-indole-	20.87	$C_{54}H_{57}N$	1119.32	1120.33	[M+H]	0.09	764.21796,602.16559,503.13364,411.10797,393.0966	ZSS	FZSS

Table S3 Detailed information of compounds identified or tentatively characterized from two seeds based on UHPLC-Q-Orbitrap-MS (continued)

NO	Compound	RT[min]	Formula	weight	[M+H]$^+$	Adduc	Error(p	MS/MS	source	
74	6'''-(-)-Phaseoylspinosin	21.18	$C_{43}H_{50}O_{19}$	870.2946	871.301<br>869.287	[M+H]<br>[M-H]$^-$	0.12<br>-0.12	327.08643,351.08673,297.07581,393.09744,429.1182<br>427.10474,292.03772,307.06149,607.16260,325.0739	ZSS	FZSS
75	6''-feruloylisopinosin	21.87	$C_{38}H_{40}O_{18}$	784.2214	785.228<br>783.215	[M+H]<br>[M-H]	0.13<br>-0.13	117.05476,447.12894,327.08640,145.02863,285.0758<br>161.02350,783.21338,175.03897,325.07074,607.1669	ZSS	FZSS
76	**Protojujuboside B***	22.8	$C_{58}H_{96}O_{2}$	1224.61	1223.60	[M-H]$^-$	-0.08	787.41345,625.36035,1223.60767,919.45612,131.033	ZSS	FZSS
77	Protojujuboside A	22.90	$C_{64}H_{106}O$	1386.66	1385.65	[M-H]$^-$	-0.07	949.46686,1081.50854,1385.66052,625.36084,787.41	ZSS	ND
78	Quercetin	23.19	$C_{15}H_{10}O_{7}$	302.042	301.035	[M-H]$^-$	-0.33	310.03549,151.00247,178.99750,107.01211,65.00166	ND	FZSS
79	Sanjoinine A	23.38	$C_{31}H_{42}N_{4}$	534.320	535.292	[M+H]	0.18	148.11227,149.11572,133.08884,105.07053,86.09711	ZSS	FZSS
80	Lotusanine A	25.02	$C_{31}H_{42}N_{4}$	534.320	535.292	[M+H]	0.18	148.11221,149.11583,133.08884,287.17566,86.09708	ZSS	FZSS
81	6''-feruloyl-6'''-	25.41	$C_{46}H_{46}O_{2}$	934.253	933.245	[M-H]$^-$	-0.11	577.13464,292.03854,235.06155,175.03925,134.0359	ND	FZSS
82	Apigenin	25.71	$C_{15}H_{10}O_{5}$	270.0528	269.045<br>630.366	[M-H]$^-$<br>[M-H]$^-$	-0.37<br>-0.98	225.05478,151.00253,117.0325,107.01212<br>342.18246,630.36768,273.12460,134.05960,247.1446	ZSS	ND
83	Amphibine-D	25.83	$C_{36}H_{49}N_{5}$	631.373	632.381	[M+H]	0.16	148.11224,289.19113,261.19626,344.19696,149.1157	ZSS	FZSS
84	6'',6'''-Diferuloylspinosin	26.28	$C_{48}H_{48}O_{21}$	960.2688	961.276<br>959.262	[M+H]<br>[M-H]$^-$	0.10<br>-0.10	177.05479,327.08652,145.02866,393.09711,351.0861<br>160.01541,175.03888,134.03581,603.15051,235.0606	ZSS	FZSS
85	**Jujuboside A***	27.06	$C_{58}H_{94}O_{2}$	1206.60	1251.60	[M-	3.73	1205.59631,1073.55420,911.50153,749.44873,603.39	ZSS	FZSS
86	Jujuboside C	27.12	$C_{59}H_{96}O_{2}$	1236.61	1281.61	[M-	3.64	1235.60669,1073.55420,1055.54431,911.49921,749.4	ZSS	FZSS
87	Jujuboside A$_1$	27.53	$C_{58}H_{94}O_{26}$	1206.6033	1251.61<br>1207.61	[M-<br>[M+H]	3.73<br>0.08	1205.59595,1073.55420,749.44928,101.02277,125.02<br>455.35229,369.28024	ZSS	FZSS
88	**Jujuboside B***	28.17	$C_{52}H_{84}O_{21}$	1044.5505	1043.54<br>1045.55	[M-H]$^-$<br>[M+H]	-0.10<br>0.10	911.50244,749.44916,101.02282,603.39124,1043.545<br>587.39655,455.35226,437.34222,391.28311,369.2785	ZSS	FZSS
89	Jujuboside Ⅱ	28.18	$C_{52}H_{84}O_{21}$	1044.5505	1089.55<br>1045.55	[M+H-<br>[M+H]	4.31<br>0.10	1043.54431,911.50189,749.44861,603.39081,161.044<br>473.34222,455.35226	ZSS<br>ND	FZSS
90	Jujuboside I	28.24	$C_{53}H_{86}O_{2}$	1074.56	1119.55	[M-	4.19	1073.55493,911.50348,749.44977,603.39270,101.022	ZSS	FZSS

Table S3 Detailed information of compounds identified or tentatively characterized from two seeds based on UHPLC-Q-Orbitrap-MS (continued)

NO	Compound	RT[min]	Formula	weight	[M+H]+	Adduc	Error(p	MS/MS	source	
91	Jujuboside B1	28.70	$C_{52}H_{84}O_{2}$<br>$_{1}$	1044.55<br>05	1043.54<br>1045.55	$[M-H]^-$<br>[M+H]	-0.10<br>0.10	1043.54504,911.50427,749.44965,603.39154,131.033<br>455.35226,437.34222	ZSS	FZSS
92	JujubosideIII	28.7	$C_{52}H_{84}O_{2}$<br>$_{1}$	1044.55<br>05	1089.54<br>1045.55	[M-<br>[M+H]	4.31<br>0.10	1043.54504,911.50427,749.44965,603.39154,131.033<br>455,35226,437.34222	ZSS<br>ND	FZSS
93	Acetyljujuboside B	29.49	$C_{54}H_{86}O_{2}$	1086.56	1085.55	$[M-H]^-$	-0.09	749.44946,1025.54199,911.50049,893.48944,1043.54	ZSS	FZSS
94	24-Hydroxyceanothic	31.25	$C_{30}H_{46}O_{6}$	502.329	501.322	$[M-H]^-$	-0.20	501.32303,471.31271,427.32397,472.31537	ZSS	FZSS
95	epiceanothic acid	33.11	$C_{30}H_{46}O_{5}$	486.334	485.327	$[M-H]^-$	-0.21	485.32736,423.32755,486.33093,60.991146	ZSS	FZSS
96	27-Hydroxyceanothic	33.25	$C_{30}H_{46}O_{6}$	502.329	501.322	$[M-H]^-$	-0.20	501.32272,471.31213,427.32193,409.31149,60.99146	ZSS	FZSS
97	16-	34.03	C_{16} H_{32}	272.235	271.228	$[M-H]^-$	-0.37	271.22800，253.21693，225.22185	ZSS	FZSS
98	**Ceanothic acid***	34.25	$C_{30}H_{46}O_{5}$	486.334	485.327	$[M-H]^-$	-0.21	485.32770,423.32788,60.99155,486.33115,424.32938	ZSS	FZSS
99	Diisobutylphthalate	34.537	C_{16} H_{22}	278.151	279.159	[M+H]	0.36	149.02362,167.03398,	ZSS	FZSS
100	Oleamide	34.895	C_{18} H_{35}	281.271	282.279	[M+H]	0.36	282.27930,265.25259,247.24216,114.09179,95.08614	ZSS	FZSS
101	Linoleic acid	35.63	$C_{18}H_{32}O_{2}$	280.240	279.232	$[M-H]^-$	-0.36	279.23296	ZSS	FZSS
102	**Betulinic acid***	35.76	$C_{30}H_{48}O_{3}$	455.353	454.344	$[M-H]^-$	-0.22	453.33832,116.92706,100.93176,180.88925,254.8503	ZSS	FZSS
103	Betulinic acid	35.87	$C_{30}H_{46}O_{3}$	454.684	453.337	$[M-H]^-$	-0.29	455.35333,116.92706,100.93176,180.88925,254.8503	ZSS	ND
104	Stearamide	35.907	C_{22} H_{43}	337.334	338.341	[M+H]	0.30	338.34177,321.31674,303.30481,212.20091,55.0542	ZSS	FZSS
105	Palmitic acid	36.259	C_{16} H_{32}	256.240	255.232	$[M-H]^-$	-0.39	255.23299，237.22209	ZSS	FZSS
106	Oleic acid	36.398	$C_{18}H_{34}O_{2}$	282.255	281.248	$[M-H]^-$	-0.36	281.24863	ZSS	FZSS
107	Bacopaside IV	14.96	$C_{41}H_{66}O_{1}$	750.455	795.454	[M+H	6.00	749.44727	ND	FZSS
108	Jujubogenin	19.22	$C_{30}H_{48}O_{4}$	472.355	473.362	[M+H]	0.21	455.35379,437.34119,419.33176,369.27817	ND	FZSS

Table S4

Table S4 Differential metabolite expression (VIP > 1.00) in ZSS and FZSS based on UHPLC-Q-Orbitrap-MS

NO.	RT (min)	Name	Molecular Formular	VIP	Molecular weight	Quasi-Molecular	Error (ppm)	[M+H]$^+$ [M-H]$^-$ [M-H+FA]$^-$	ZSS	FZSS	Type
49	13.86	**Spinosin***	$C_{28}H_{32}O_{15}$	9.542	608.1741	609.1821	0.0007	[M+H]$^+$	ZSS	FZSS	Flavonols
2	36.91	Stearamide	$C_{18}H_{37}NO$	8.382	284.2950	337.3344	0.730	[M+H]$^+$	ZSS	FZSS	Fatty acid derivative
68	18.48	**6'''-Feruloylspinosin***	$C_{38}H_{40}O_{18}$	6.610	784.2215	785.2286	-0.0001	[M+H]$^+$	ZSS	FZSS	Flavonols
32	9.48	**Coclaurine***	$C_{17}H_{19}NO_3$	6.048	285.1365	286.1440	-0.0002	[M+H]$^+$	ZSS	FZSS	Nitrogen-containig compounds
5	1.566	Choline	$C_5H_{13}NO$	5.890	103.0997	104.1075	4.754	[M+H]$^+$	ZSS	FZSS	amino acid and amino acid derivative
41	12.98	Isovitexin-2''-*O*-*β*-glucopyranoside	$C_{27}H_{30}O_{15}$	5.516	594.1585	595.16652	0.140	[M+H]$^+$	ZSS	FZSS	Flavonols
2	1.50	Arginine	$C_6H_{14}N_4O_2$	3.586	174.1117	175.1193	3.734	[M+H]$^+$	ZSS	FZSS	amino acid and amino acid derivative
20	3.54	Betaine	$C_5H_{11}NO_2$	6.941	117.0790	118.0867	3.543	[M+H]$^+$	ZSS	FZSS	Nitrogen-containig compounds
99	34.54	Diisobutylphthalate	$C_{16}H_{22}O_4$	5.207	278.1520	278.1518	0.710	[M+H]$^+$	ZSS	FZSS	Fatty acid derivative
100	34.90	Oleamide	$C_{18}H_{35}NO$	6.864	282.2793	281.2718	0.961	[M+H]$^+$	ZSS	FZSS	Fatty acid derivative
102	35.76	**Betulonic acid***	$C_{30}H_{46}O_3$	4.769	455.3531	454.3446	0.02	[M-H]-	ZSS	FZSS	saponin
98	34.25	**Ceanothic acid***	$C_{30}H_{46}O_5$	4.112	486.3345	485.3272	0.000	[M-H]$^-$	ZSS	FZSS	saponin
52	14.20	**Isovitexin***	$C_{21}H_{20}O_{10}$	3.676	432.1056	433.1135	0.0004	[M+H]$^+$	ZSS	FZSS	Flavonols
95	33.11	**epiceanothic acid***	$C_{30}H_{46}O_5$	3.290	486.3345	485.3272	0.0003	[M-H]-	ZSS	FZSS	saponin
34	10.00	**Vicenin II***	$C_{27}H_{30}O_{15}$	3.174	594.1585	595.1657	-0.0207	[M+H]$^+$	ZSS	FZSS	Flavonols

Table S4 Differential metabolite expression (VIP > 1.00) in ZSS and FZSS based on UHPLC-Q-Orbitrap-MS(continued)

NO.	RT (min)	Name	Molecular Formular	VIP	Molecular weight	Quasi-Molecular	Error (ppm)	$[M+H]^+$ $[M-H]^-$ $[M-H+FA]^-$	ZSS	FZSS	Type
46	13.69	Isospinosin	$C_{28}H_{32}O_{15}$	3.025	608.1741	609.1821	0.0007	$[M+H]^+$	ZSS	FZSS	Flavonols
60	16.70	Isovitexin-2''-*O*-(6-p-coumaloyl)-glucopyranoside	$C_{36}H_{36}O_{17}$	2.685	740.1952	741.2035	0.001	$[M+H]^+$	ZSS	FZSS	Flavonols
54	14.88	**Swertisin***	$C_{22}H_{22}O_{10}$	2.681	446.1213	447.1286	-0.0004	$[M+H]^+$	ZSS	FZSS	Flavonols
19	18.29	6'''-p-coumaroylspinosin	$C_{37}H_{38}O_{17}$	2.574	754.2109	755.2190	0.0007	$[M+H]^+$	ZSS	FZSS	Flavonols
67	13.27	Kaempferol3-*O*-beta-glucopyranoside-7-*O*-alpha-rhamnopyranoside	$C_{27}H_{30}O_{15}$	2.38	594.1584	595.16652	0.001	$[M+H]^+$	ZSS	FZSS	Flavonols
58	16.06	Kaempferol-3-*O*-rutinoside	$C_{27}H_{30}O_{15}$	2.349	594.1585	595.1666	1.311	$[M+H]^+$	ZSS	FZSS	Flavonols
48	13.83	Kaempferol	$C_{15}H_{10}O_6$	2.306	286.0477	287.0553	1.022	$[M+H]^+$	ZSS	FZSS	Flavonols
33	9.89	Juzirine	$C_{17}H_{15}NO_3$	2.398	281.1052	282.1125	0.0001	$[M+H]^+$	ZSS	FZSS	Nitrogen-containig compounds
35	10.01	Norisocorydine	$C_{19}H_{21}NO_4$	2.278	279.1623	328.1543	0.000	$[M+H]^+$	ZSS	FZSS	Nitrogen-containig compounds
26	7.25	N-glc-indoleaceticaid	$C_{23}H_{29}NO_8$	2.078	447.1893	448.1966	-0.115	$[M+H]^+$	ZSS	FZSS	Nitrogen-containig compounds
57	16.02	6'''-Vanilloylspinosin	$C_{36}H_{38}O_{18}$	2.127	758.2058	759.2140	0.0009	$[M+H]^+$	ZSS	FZSS	Flavonols
62	16.85	Nervilifordin J	$C_{37}H_{38}O_{18}$	2.052	770.2058	771.2140	4.533	$[M+H]^+$	ZSS	FZSS	Flavonols
10	2.057	Citric acid	$C_6H_8O_7$	2.063	192.0270	283.2643	0.161	$[M-H]^-$	ZSS	FZSS	organic acid
88	28.17	**Jujuboside B***	$C_{52}H_{84}O_{21}$	2.020	1044.5505	1089.5446	1.675	$[M-H+FA]^-$	ZSS	FZSS	saponin
38	11.83	Lotusine	$C_{19}H_{24}NO_3^+$	1.955	315.1828	314.1390	-1.242	$[M+H]^+$	ZSS	FZSS	Nitrogen-containig compounds
47	13.78	Caaverine	$C_{17}H_{17}NO_2$	1.942	268.1332	268.1334	-0.029	$[M+H]^+$	ZSS	FZSS	Nitrogen-containig compounds

Table S4 Differential metabolite expression (VIP > 1.00) in ZSS and FZSS based on UHPLC-Q-Orbitrap-MS (continued)

NO.	RT (min)	Name	Molecular Formular	VIP	Molecular weight	Quasi-Molecular	Error (ppm)	$[M+H]^+$ $[M-H]^-$ $[M-H+FA]^-$	ZSS	FZSS	Type
65	17.94	6'''-Sinapoylspinosin	$C_{39}H_{42}O_{19}$	1.898	814.2320	815.2405	0.0012	$[M+H]^+$	ZSS	FZSS	Flavonols
83	25.83	Amphibine-D	$C_{36}H_{49}N_5O_5$	1.723	632.3806	632.3814	-0.001	$[M+H]^+$	ZSS	FZSS	Nitrogen-containig compounds
15	2.55	Adenine	$C_5H_5N_5$	1.628	135.0545	136.0620	-9.920	$[M+H]^+$	ZSS	FZSS	amino acid and amino acid derivative
85	27.06	**Jujuboside A***	$C_{58}H_{94}O_{26}$	1.581	1206.6033	1251.6078	1.192	$[M-H+FA]^-$	ZSS	FZSS	saponin
63	17.74	Azelaic acid	$C_9H_{16}O_4$	1.574	188.1049	187.0966	-5.100	$[M-H]^-$	ZSS	FZSS	organic acid
43	13.26	**Magnoflorine***	$C_{20}H_{24}NO_4$	1.415	343.1778	342.1700	0.0001	$[M+H]^+$	ZSS	FZSS	Nitrogen-containig compounds
38	10.11	Catechin	$C_{15} H_{14} O_6$	1.396	290.0790	291.0719	0.565	$[M+H]^+$	ZSS	FZSS	Flavonols
75	21.87	6''-feruloylisopinosin	$C_{38}H_{40}O_{18}$	1.179	784.2215	785.2286	4.533	$[M+H]^+$	ZSS	FZSS	Flavonols
22	6.52	Tryptophan	$C_{11}H_{12}N_2O_2$	1.145	204.0899	205.0975	1.645	$[M+H]^+$	ZSS	FZSS	amino acid and amino acid derivative
70	20.00	6''-*O*-(3-glc-indole-acetyl)spinosin(R)	$C_{44}H_{49}NO_{22}$	1.083	943.2746	944.2830	-0.0009	$[M+H]^+$	ZSS	FZSS	Flavonols
24	6.787	5-Hydroxyindole-3-acetic acid	$C_{10}H_9NO_3$	1.083	191.0582	192.0658	1.581	$[M-H]^-$	ZSS	FZSS	organic acid
14	2.48	Guanine	$C_5H_5N_5O$	1.059	151.0494	152.0570	1.962	$[M+H]^+$	ZSS	FZSS	amino acid and amino acid derivative

Table S5

Table S5 Calibration curve data for quantitative analysis based on GC-MS

Compound	quantitative	Standard calibration	r	linearity range (μg mL^{-1})
palmitic acid	117	Y=0.3705X-0.1357	0.9971	8.35-870.98
stearic acid	145.1	Y=0.2822X-0.0904	0.9900	8.12-442.57
eicosanoic acid	116.1	Y=0.1032X-0.0015	0.9993	11.18-204.08
squalene	81.1	Y=0.3985X-0.1070	0.9965	25.22-1403.75
9-Octadecenoic acid	96.1	Y=0.1996X-0.0045	0.9999	70.60-2896.39
γ-tocopherol	223.1	Y=0.2600X-0.0069	0.9970	12.75-809.48
9,12-Octadecadienoic acid	135	Y=0.3417X-0.0225	0.9992	23.49-870.98

Table S6

Table S6 Calibration curve data for quantitative analysis based on HPLC

Compound	Standard calibration curves	r	linearity range (μg mL^{-1})
magnoflorine	Y=5170X-131139	0.9991	3.2-320
coclaurine	Y=29654X-32542	0.9994	0.8-80
Vicenin II	Y=26588X-6948.3	0.9993	0.6-20
spinosin	Y=21887X-24139	0.9994	2-200
6‴- feruloylspinosin	Y=25273X-29683	0.9994	1.8-180
jujuboside A	Y=1.2737X-3.2302	0.9970	10-160
jujuboside B	Y=1.2533X+3.1018	0.9992	10-100

Table S7

Table S7 The content of seven fatty acid compositions

Compound	ZSS (mg/g)	FZSS (mg/g)
palmitic acid	2.46±0.10	0.88±0.19***
9,12-Octadecadienoic acid	17.45±0.22	7.82±0.21***
9-Octadecenoic acid	20.33±0.26	6.51±0.24***
stearic acid	1.28±0.02	0.46±0.11***
eicosanoic acid	0.30±0.02	0.15±0.04***
squalene	0.82±0.03	0.91±0.57
γ-tocopherol	0.87±0.03	0.99±0.23
total fatty acid	43.68±0.52	18.94±0.66***

*$p < 0.05$, ** $p < 0.01$, *** $p < 0.001$

Table S8

Table S8 The content of seven secondary metabolic components.

Compound	ZSS (mg/g)	FZSS (mg/g)
magnoflorine	1.21 ± 0.05	1.46 ± 0.02***
coclaurine	0.14 ± 0.01	0.17 ± 0.01**
Vicenin II	0.03 ± 0.00	0.04 ± 0.00
spinosin	0.49 ± 0.02	0.56 ± 0.01***
6‴- feruloylspinosin	0.76 ± 0.02	0.91 ± 0.02***
jujuboside A	0.56 ± 0.03	0.67 ± 0.01**
jujuboside B	0.19 ± 0.02	0.14 ± 0.01**

*$p < 0.05$, ** $p < 0.01$, *** $p < 0.001$
